# Decoding stakeholders' demand to map the future of smart communities: evidence from China

**DOI:** 10.3389/fpubh.2026.1751235

**Published:** 2026-03-13

**Authors:** Wei Qi, Yujia Shan, Ling Ma, Hao Wu, Hongtu Yan, Pengju Han, Tiantian Gu

**Affiliations:** 1 School of Construction Management, Jiangsu Vocational Institute of Architectural Technology, Xuzhou, China; 2 School of Mechanics and Civil Engineering, China University of Mining and Technology, Xuzhou, China

**Keywords:** cluster analysis, community governance, community resilience, smart community, stakeholder demand

## Abstract

**Background:**

Smart community development is critical for enhancing community resilience and strengthening collective capacities to respond to public health and other urban crises. A model-based taxonomy of stakeholder demands remains absent, as existing exploration has largely been limited to analyses of single stakeholder groups.

**Method:**

This study therefore develops a comprehensive classification model to capture stakeholder demands across three critical dimensions: community safety, livability services, and community governance. By integrating hierarchical clustering and K-means analysis of survey data from 1,606 respondents across 32 typical smart communities in China, a clear taxonomy of stakeholder demand emerges.

**Results and Discussion:**

The analysis identifies four distinct clusters: Cluster 1 (strong livability services demand), Cluster 2 (prioritized safety and governance demand), Cluster 3 (comprehensive demand across all dimensions), and Cluster 4 (weak livability-focused demand). Furthermore, these clusters reveal a consistent evolutionary pathway along a low–medium–high demand continuum.

**Conclusion:**

The findings provide a basis for public health and crisis governance, enabling targeted strategies that address differentiated community demands and foster more resilient communities.

## 1 Introduction

Smart community development is crucial in advancing sustainable urbanization and strengthening community resilience, forming the operational foundation of smart city strategies (1). These initiatives leverage digital technologies worldwide to improve urban livability, efficiency, and governance (2). Beyond these routine benefits, the same digital infrastructure proved its value during recent public health emergencies by enabling rapid case identification and effective community containment, while also ensuring efficient allocation of critical resources (3). However, the success of these endeavors depends critically on the complex interplay among key stakeholders–including residents, property service enterprises, public administrators, and social organizations (4). Each group possesses distinct priorities and expectations, and failing to systematically reconcile these multi-faceted demands can result in misaligned investments, low adoption rates, and project failures (5, 6). Therefore, a thorough understanding of stakeholder demands is essential for shifting smart community development from a technology-centric to a human-centric paradigm, which is vital for fostering the social cohesion required in public health and other crisis contexts (7).

In China, smart community development has become a national priority, recognized as a vital mechanism for modernizing grassroots governance and social management (8). Driven by top-down policy directives, widespread implementation of intelligent infrastructure and digital platforms has been observed nationwide (9). Nevertheless, this rapid expansion has often revealed a disconnect between policy design and practical outcomes, which is reflected in the inconsistent satisfaction levels among different stakeholder groups (10). The COVID-19 pandemic illustrated this clearly by activating a spectrum of concurrent demands across all stakeholders, ranging from the need for infection alerts and disinfection protocols to support for vulnerable populations (11). This implementation gap underscores a pressing need to replace the prevalent, uniform approach with a more nuanced strategy. A foundational step toward this goal is the systematic identification and classification of the heterogeneous demands of all four key stakeholder groups, which is a prerequisite for designing tailored and effective community development patterns (12).

Recent research on smart communities has increasingly focused on the operational efficacy of core functions and collaborative processes among stakeholders. This focus aligns with Sustainable Development Goal 11 (SDG11), which prioritizes inclusive, safe, and resilient urban development (13). Extensive exploration has been conducted regarding community demands and stakeholder engagement. This research has demonstrated that smart communities depend not only on technological infrastructure, but also on the integration of multi-dimensional functional goals and meaningful stakeholder engagement (14, 15). Nevertheless, the scholarly discourse remains markedly fragmented. A substantial body of research has been dedicated to examining the demands of single stakeholder groups in isolation (16). For instance, the priorities of residents have been studied, often highlighting concerns related to service convenience, safety, and community engagement (17, 18). Concurrently, the perspectives of property service enterprises have been analyzed, focusing on their operational efficiency and business model innovation in the digital era (16). Similarly, the role of public administrators has been investigated, emphasizing policy implementation and governance efficacy (19, 20). Although these studies offer detailed insights into the priorities of specific stakeholders, there was limited integration of the stakeholder demands for collaborative governance. Moreover, beyond studies focused on individual stakeholders, another set of research has centered on participatory governance frameworks in specific service areas, such as smart healthcare or older adult care services (21, 22).

However, these frameworks mostly address the mechanisms of stakeholder engagement rather than the systematic analysis of different demands into a unified taxonomy. Consequently, there's still gaps between the recognized imperative for multi-actor collaboration and the lack of a comprehensive demand that incorporates all stakeholder perspectives. This collective body of work, while valuable, reveals three interconnected gaps. First, a comprehensive demand indicator system that systematically integrates the perspectives of all four key stakeholder groups into a unified architecture has not been established. Second, robust quantitative methodologies for objectively classifying the multi-dimensional demand profiles are notably underutilized. Third, the potential evolutionary pathways between different demand clusters remain largely unexamined, thereby overlooking the dynamic nature of stakeholders' needs for smart community development.

To address these gaps, this study constructs a comprehensive stakeholder demand indicator system for smart community development and develops a quantitative classification model by integrating hierarchical clustering and K-means analysis. Applied to a substantial dataset of 1,606 validated survey responses, this methodology enables the empirical identification of distinct stakeholder demand clusters while facilitating examination of their interrelationships. Theoretically, this research contributes a data-driven taxonomy to the field, shifting the analytical paradigm from isolated examinations to a holistic understanding of integrated demand structures. Practically, the findings offer clear guidance for customizing development strategies to specific demand profiles, thereby enhancing the efficacy of smart community initiatives and strengthening community resilience through digital services that are dynamically aligned with evolving stakeholder priorities during crises.

## 2 Methodology

### 2.1 Establishment of the stakeholders' demand indicator system

The identification of stakeholder demand indicators was guided by the integration of Maslow's Hierarchy of Needs Theory and the Kano Model, which served as the primary conceptual foundations (23). Prior research has typically examined residents, property enterprises, public administrators, or social organizations separately, using frameworks tailored to specific stakeholder groups or community contexts. By contrast, these two theories offer both the necessary generality and the capacity for differentiation to capture demands across all four stakeholder groups (24). Maslow's Hierarchy provides a general developmental structure from safety through self-actualization, making it applicable to all stakeholders regardless of their institutional role (25). Meanwhile, the Kano Model explains why different stakeholders classify the same service attributes into different quality categories (26). For example, public administrators may classify community grid-based governance as “Must-be” (threshold requirement), whereas residents may regard the same functions as “Attractive” (enhancement quality). This integrated framework enables a comprehensive analysis of stakeholders' demands spanning from fundamental safety to community governance.

**Figure 1:**
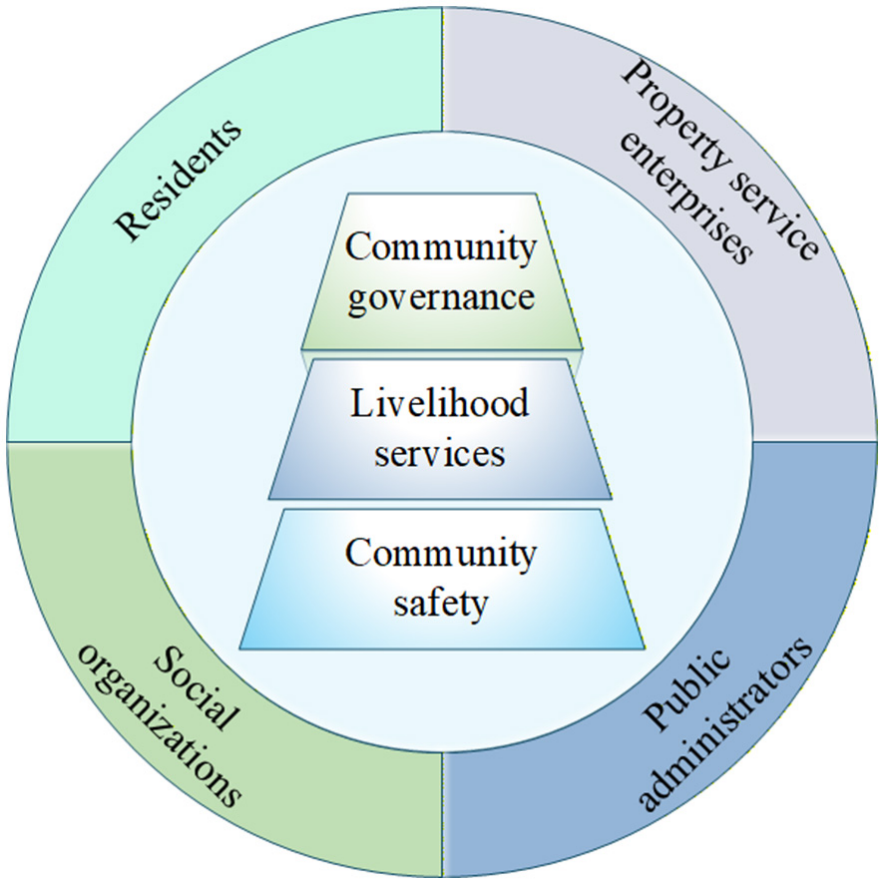
Stakeholders' demand framework for smart community development.

Within this integrated framework (see Figure 1), three core dimensions of stakeholder demands were derived. The first dimension, Community Safety, aligns with the “safety needs” level in Maslow's hierarchy, reflecting stakeholders' fundamental requirements for the protection of life and property (27). In the Kano Model, these correspond to “Must-be Quality” attributes, whose absence leads to significant dissatisfaction (27). The second dimension, Livability Services, relates to the “love and belongingness” and “esteem needs” in Maslow's theory, encompassing services that enhance quality of life and social identity (28). These are categorized largely as “One-dimensional Quality” demands in the Kano Model, where satisfaction increases with better service performance (27). The third dimension, Community Governance, connects to the “self-actualization needs” in Maslow's hierarchy, involving stakeholder participation in community decision-making. These represent “Attractive Quality” attributes in the Kano Model, which can greatly enhance satisfaction when present but do not cause dissatisfaction if absent (26).

An initial set of 65 secondary indicators across these three dimensions was identified through a systematic review of 88 relevant studies, supplemented by insights from the theoretical framework. This preliminary demand indicator system is provided in Supplementary File S1. Subsequently, to select the appropriate demand indicators, an expert scoring process was conducted with 38 valid responses from a panel of 50 invited experts, comprising both academics and experienced practitioners (see Table 1 for expert demographics). Each indicator was rated on a five-point Likert scale for importance.

**Table 1:** Basic information of the 38 experts surveyed.

**Variable**	**Items**	**Percentage (%)**
Gender	Male	47.37
	Female	52.63
Years of experience	Over 5 years	18.42
	4–5 years	31.58
	1–3 years	23.68
	Within 1 year	26.32
Professional field	Academia	39.47
	Enterprise	60.53
Professional title	Doctorate	31.58
	Master's degree	44.74
	Bachelor's degree	18.42
	Others (high school and below)	5.26

The weighted average method of membership analysis within fuzzy set theory was then applied to determine the membership degree *R*_*t*_ of each indicator using the formula (29):


$$\begin{array}{c} R_{t}=\frac{P_{t}+0.5\;*\;{P_{t}}^{\prime }}{P} \end{array}$$ (1)

where *P*_*t*_ represents the number of experts rating the *t*-th indicator as “very important,” ${P_{t}}^{\prime }$ denotes those rating it as “important,” and *P* is the total number of experts surveyed. Indicators with a membership degree *R*_*t*_≥0.5 were retained, while those below this threshold were excluded. This retention threshold represents the critical point at which the weighted proportion of experts rating an indicator as “important” or “very important” exceeds 50% of the panel, thereby satisfying the majority consensus criterion (30). Furthermore, to ensure the external validity of the indicator system, experts were asked to participate in the validation exercise for external validity of the indicator system. Of these, 34 experts (89.5%) fully endorsed the proposed dimensional structure without modification, indicating strong consensus regarding the conceptual boundaries and operational definitions of the three primary dimensions. Four experts (10.5%) provided specific recommendations for indicator reclassification or consolidation to enhance discriminant validity and reduce conceptual overlap. Detailed information on the questionnaire of demand indicators and the corresponding results is provided in Supplementary File S2. This process resulted in the final demand indicator system, consisting of three primary and 45 secondary indicators, as summarized in Table 2. The specific details of these demand indicators can be found in Supplementary File S3.

**Table 2:** The final stakeholders' demand indicator system for smart community development.

**Dimension**	**ID**	**Indicators**	**References**
Community safety	Y1-1	Digital community emergency preparedness plan	(37, 68, 84–87)
	Y1-2	Emergency plan implementing	
	Y1-3	Propaganda and education of emergency safety	
	Y1-4	Abnormal events recording	
	Y1-5	Management and control of key parts	
	Y1-6	Building monitoring	
	Y1-7	Life channel facilities monitoring	
	Y1-8	Floating population management	
	Y1-9	Smart object monitoring facilities	
	Y1-10	Public facilities monitoring	
	Y1-11	Smart firefighting facilities	
	Y1-12	Community safety inspection	
	Y1-13	Emergency duty	
	Y1-14	Intelligent emergency alert and forecasting	
	Y1-15	Coordinated emergency response	
	Y1-16	Emergency broadcast system	
	Y1-17	Emergency rescue alarm	
	Y1-18	Emergency supplies reserve	
	Y1-19	Emergency command and dispatch	
	Y1-20	Post-response community safety evaluation	
Livability services	Y2-1	Community service center	(69, 88–92)
	Y2-2	Community self-service terminals	
	Y2-3	Community health services	
	Y2-4	Community medical services	
	Y2-5	Intelligent older adult care services	
	Y2-6	Centralized reporting & maintenance system	
	Y2-7	Recycling system for used things	
	Y2-8	Smart childcare	
Community governance	Y3-1	Community grid-based governance	(19, 43, 51, 93, 94)
	Y3-2	Collaborative community governance	
	Y3-3	Smart environment monitoring	
	Y3-4	Community population management	
	Y3-5	Community vehicle management	
	Y3-6	Community party affairs management	
	Y3-7	Community volunteer management	
	Y3-8	Housing management	
	Y3-9	Integrated government service system	
	Y3-10	Support services for vulnerable groups	
	Y3-11	Dispute mediation and legal outreach	
	Y3-12	Community cultural and recreational activities	
	Y3-13	Centralized incident dispatch and monitoring	
	Y3-14	Community alert broadcasting and statistics	
	Y3-15	Multi-sectoral linkage	
	Y3-16	Monitoring of special population groups	
	Y3-17	Information management for vulnerable groups	

### 2.2 Development of the stakeholders' demand classification model

**Figure 2:**
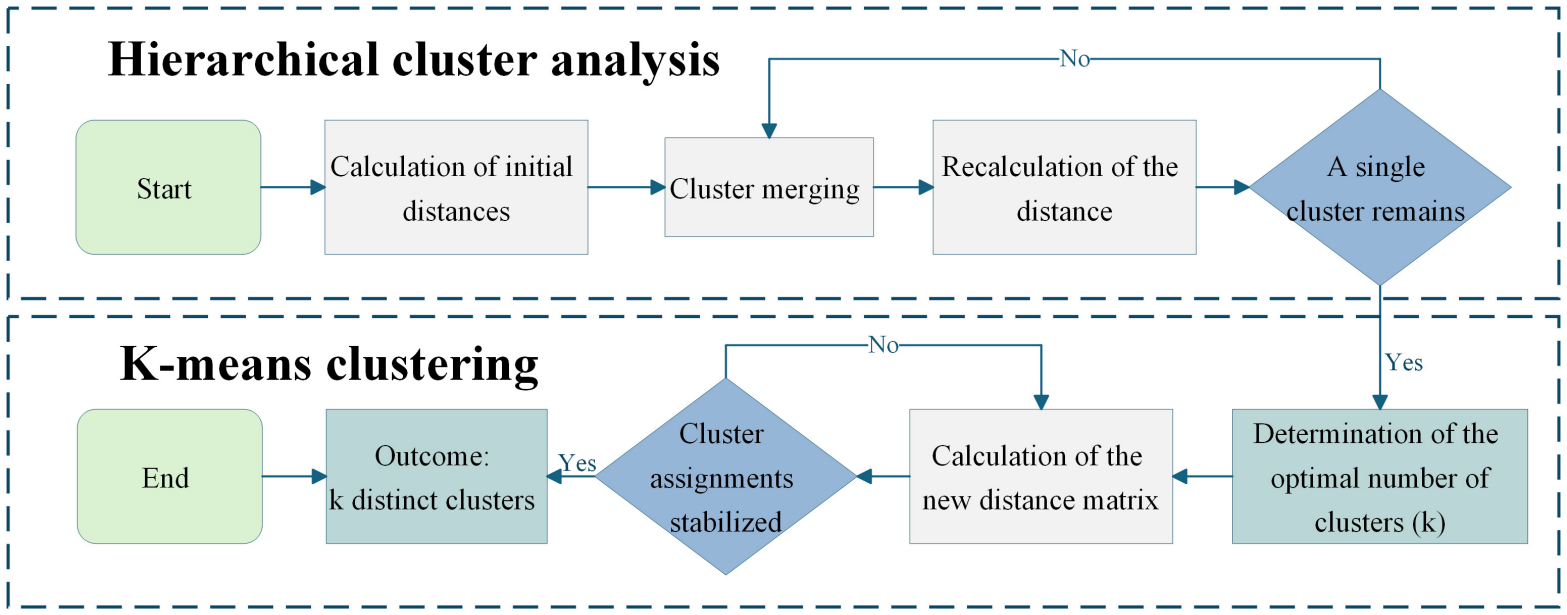
Analytical process of the stakeholders' demand classification model.

Following the establishment of the stakeholder demand indicator system, a demand classification model was developed utilizing cluster analysis. Cluster analysis, as a multivariate statistical technique, enables the grouping of samples based on similarity measures, making it particularly suitable for identifying typologies in complex stakeholder demand data (31). Specifically, a demand classification model integrating hierarchical cluster analysis (HCA) and k-means clustering was developed to systematically categorize stakeholder demand profiles. This integration was selected to leverage the complementary strengths of both methods: HCA enables the determination of the optimal cluster number without prior specification through analysis of the agglomeration schedule, whereas K-means provides computational efficiency and stable cluster assignment for large-sample survey data (32). The overall analytical process is illustrated in Figure 2.

(a) Calculation of initial distances

The analysis commenced by computing the average scores of the dimensions of community safety, livelihood services, and community governance, which subsequently served as the input data for the clustering procedure. Subsequently, the initial configuration set the number of clusters equal to the number of samples. In the hierarchical clustering phase, the squared Euclidean distance (denoted as *d*_*ij*_) and the sum of squared deviations *S* were calculated using the following formula:


$$\begin{array}{ccc} d_{ij} & = & \overset{m}{\underset{t=1}{\sum }}{\left(x_{it}-x_{jt}\right)}^{2} \end{array}$$ (2)

$$\begin{array}{l} S=\sum_{i=1}^{n}{\left(x_{i1}-\frac{\sum_{i=1}^{n}x_{i1}}{n}\right)}^{2}+\sum_{i=1}^{n}{\left(x_{i2}-\frac{\sum_{i=1}^{n}x_{i2}}{n}\right)}^{2}+...\\ \;+\sum_{i=1}^{n}{\left(x_{im}-\frac{\sum_{i=1}^{n}x_{im}}{n}\right)}^{2} \end{array}$$ (3)

where *d*_*ij*_ denotes the distance between the *i*-th sample and the *j*-th sample in terms of three dimensions. *x*_*it*_ and *x*_*jt*_ represent the data of the *t*-th indicator in the *i*-th sample and *j*-th sample, respectively. As all demand indicators were measured using a uniform five-point Likert scale (ranging from 1 = “strongly unnecessary” to 5 = “strongly necessary”), the variables shared identical metric properties and units of measurement. Therefore, data standardization was not applied, as z-score transformation is unnecessary when all variables share the same scale and range.

(b) Cluster merging based on minimum distance

The sum of squared deviations *S* was minimized at each merging step. When clusters *p* and *q* are merged into a new cluster *l*, the sum of squared deviations of cluster *l* and the distance between clusters *p* and *q* (*d*_*pq*_) was then calculated as:


$$\begin{array}{ccc} S_{l} & = & S_{p}+S_{q}+\frac{n_{p}n_{q}}{n_{p}+n_{q}}{\left(\bar{x_{p}}-\bar{x_{q}}\right)}^{2} \end{array}$$ (4)

$$\begin{array}{ccc} d_{pq} & = & △S_{pq}=\frac{n_{p}n_{q}}{n_{p}+n_{q}}{\left(\bar{x_{p}}-\bar{x_{q}}\right)}^{2} \end{array}$$ (5)

where *p* and *q* contain *n*_*p*_ and *n*_*q*_ samples and their corresponding sums of squared deviations are denoted as *S*_*p*_ and *S*_*q*_.

(c) Recalculation of the distance between the new cluster and all other clusters

The distance between the new cluster *l* and any arbitrary cluster *r* was calculated using the following formula:


$$\begin{array}{c} d_{lr}=\frac{n_{p}+n_{r}}{n_{l}+n_{r}}d_{pr}+\frac{n_{q}+n_{r}}{n_{l}+n_{r}}d_{qr}-\frac{n_{r}}{n_{l}+n_{r}}d_{pq} \end{array}$$ (6)

(d) Determination of the optimal number of clusters (k).

Steps (b) and (c) were repeated iteratively to merge the two closest clusters until a single cluster remains. This iterative process yields the agglomeration coefficients for different numbers of clusters. The agglomeration coefficient, which quantifies the dissimilarity level at which two clusters are merged, was examined for solutions ranging from *k* = 2 to *k* = 15. The optimal number of clusters (*k*) was determined by identifying the inflection point at which the incremental increase in the agglomeration coefficient declined sharply (30).

(e) Calculation of the new distance matrix

Using the optimal number of clusters (*k*) determined previously, k cluster centers are selected ($c_{j^{\prime }}$, where *j'*=*1, 2, …, k*). The Euclidean distance $D\left(x_{i},c_{j^{\prime }}\right)$ between each sample and every center is calculated. The distance matrix was calculated as follows:


$$\begin{array}{c} D=\left(d_{ij}\right)=\left[\begin{array}{cccc} d_{11}, & d_{12}, & ..., & d_{1k}\\ d_{21}, & d_{22}, & ... & d_{2k}\\ ... & ... & ... & ....\\ d_{n1}, & d_{n2}, & ... & d_{nk} \end{array}\right] \end{array}$$ (7)

Each sample is then assigned to the cluster *j'* with the min $D\left(x_{i},c_{j^{\prime }}\right)$, which satisfied: $D\left(x_{i},c_{j^{\prime }}\right)=\min\;\left\{D\left(x_{i},c_{j^{\prime }}\right)\right\}$ (*i* = 1, 2, …, *n*; *j'* = 1, 2, …, *k*), the sample belongs to the *j'*-th cluster.

(f) Calculation of the k new cluster centers:

$$\begin{array}{c} C_{j^{\prime }}\left(I+1\right)=\frac{1}{n_{j^{\prime }}}\underset{x^{\prime }\in C_{j^{\prime }}}{\sum }x^{\prime } \end{array}$$ (8)

where $n_{j^{\prime }}$ denotes the number of samples in cluster $C_{j^{\prime }}$; *x*′ denotes the samples classified into cluster $C_{j^{\prime }}$.

This process continued until cluster assignments stabilized and the sum of squared errors reached convergence. The final output comprised k distinct clusters, each characterized by a unique profile of stakeholder demands across the three core dimensions, thereby establishing a typology of smart community development patterns based on empirically derived demand structures.

### 2.3 Questionnaire design

To systematically obtain the demands of the four key stakeholder groups—residents, property service enterprises, public administrators, and social organizations—tailored questionnaires were developed (see Supplementary File S4). Each questionnaire was structured into three coherent parts designed to gather both quantitative ratings and qualitative insights.

The first part collected essential demographic and contextual information specific to each stakeholder type. The resident questionnaire covered characteristics such as gender, age, education level, duration of residence, and income. For property service enterprises, items included staff size, years of working in the property sector, and average monthly income. The public administrator version captured gender, age, and education level, while the social organization questionnaire recorded the respondent's position, type of organization, and years of involvement in social organization work.

The second part formed the core of the survey, measuring the perceived importance of each indicator within the three predefined dimensions: community safety, livability services, and community governance. Respondents rated their level of need for each item using a five-point Likert scale [(33); 1 = “strongly unnecessary,” 2 = “unnecessary,” 3 = “neutral,” 4 = “necessary,” and 5 = “strongly necessary”]. This structured approach enabled the quantitative assessment of demand intensity across all 45 indicators.

The third part concluded with an open-ended question, inviting additional suggestions related to smart community development. This optional component allowed respondents to express needs or perspectives not fully captured by the fixed indicators, providing valuable qualitative context to complement the quantitative data.

## 3 Study area and data collection

### 3.1 Study area

The study was conducted in six Chinese cities selected from two strategically important regions: the Guangdong-Hong Kong-Macao Greater Bay Area and cities along the Belt and Road Initiative. The Greater Bay Area was represented by Shenzhen, Dongguan, and Huizhou city, which exemplify a development model driven by technological innovation and market forces (34). Meanwhile, the Belt and Road Initiative cities of Zhengzhou, Luoyang, and Putian demonstrate a policy-guided approach characterized by strong governmental support and comprehensive digitalization of public services (35). All six cities have implemented smart community initiatives with notable achievements in technological application and service delivery (36).

**Figure 3:**
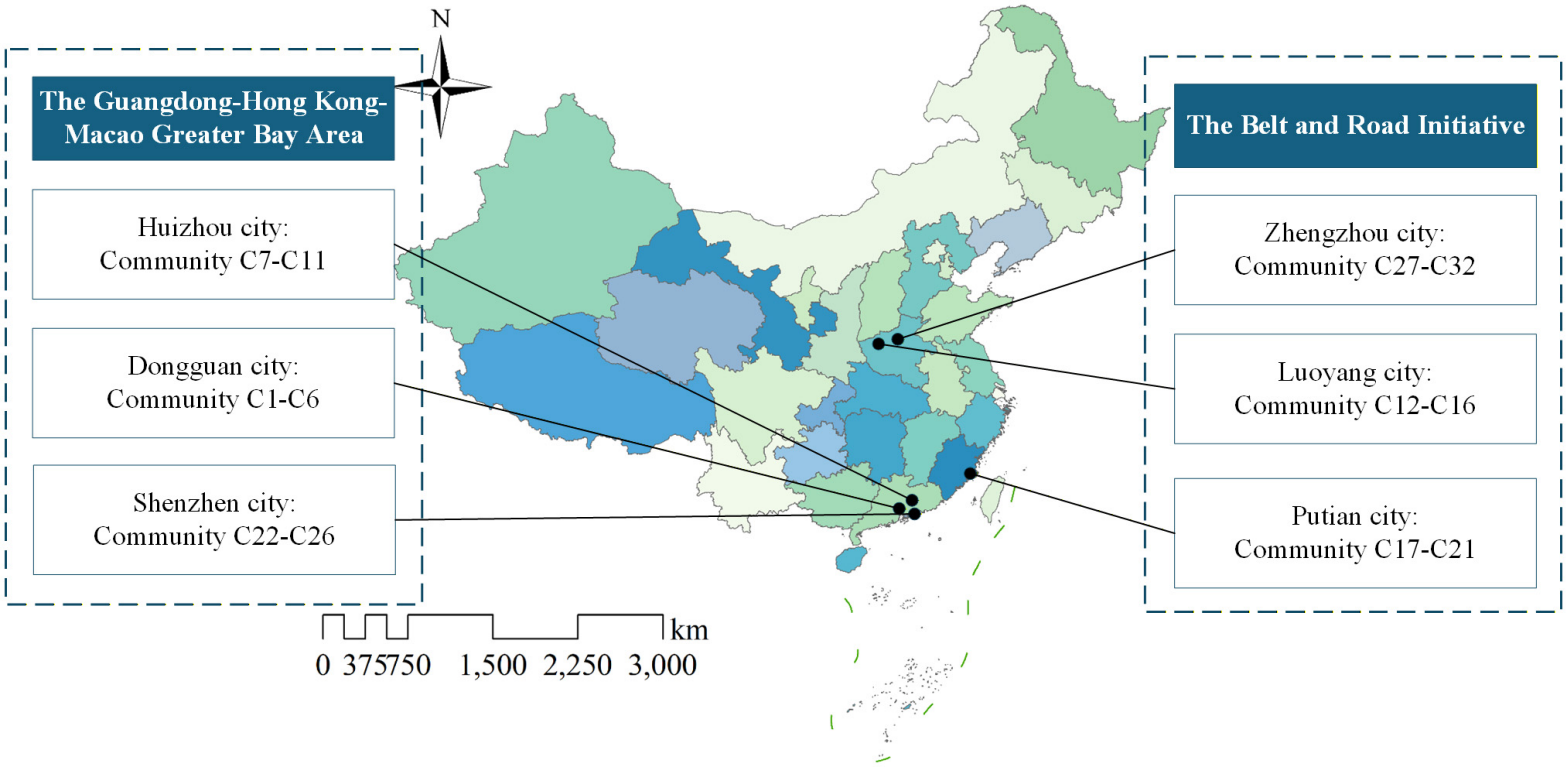
Distribution of communities surveyed for smart community construction.

Within these six cities, a total of 32 typical smart communities were identified as survey locations. These communities have been officially recognized as pilot projects with demonstrated achievements in smart community development, particularly in the domains of community safety, livability services, and governance (37). However, the emphasis of smart community development varies across the sampled sites. Specifically, communities C1-C6, C7-C11, and C22-C26 place greater emphasis on community governance, communities C17-C21 primarily focus on community safety, while communities C12-C16 and C27-C32 prioritize livelihood services. This heterogeneity suggests that, although all sampled communities have been officially designated as “smart,” their development remains uneven across functional domains. Additionally, the selection of these communities was supported by the high levels of engagement and awareness observed among key stakeholder groups, which facilitated efficient data collection and in-depth analysis of multi-stakeholder demands. The spatial distribution of the selected communities is presented in Figure 3, with full names and coding details provided in Supplementary File S5.

### 3.2 Data collection

Data collection was conducted through “Wenjuanxing,” a professional online survey platform specializing in questionnaire distribution and data collection. Utilizing this platform, a total of 2,000 questionnaires were distributed to the four stakeholder groups across the 32 selected smart communities. Following a standardized data validation process, 1,606 complete and valid responses were obtained, resulting in a response rate of 80.3%.

The valid responses showed balanced geographic distribution across the six cities: Zhengzhou (269), Luoyang (266), Putian (271), Shenzhen (266), Dongguan (264), and Huizhou (270). The sample also demonstrated strong representation across stakeholder groups, with 1,132 responses from residents, 262 from property service enterprises, 103 from public administrators, and 109 from social organizations. This comprehensive dataset enables robust analysis of multi-stakeholder demands. The detailed distribution of respondents is presented in Table 3, and the complete statistics of respondents are available in Supplementary File S6.

**Table 3:** Distribution of valid survey responses across stakeholder groups and cities.

**Stakeholder**	**Zhengzhou**	**Luoyang**	**Putian**	**Shenzhen**	**Dongguan**	**Huizhou**	**Total**
**City**						
Residents	187	190	192	186	186	191	1,132
Property service enterprises	45	42	45	42	43	45	262
Public administrators	17	16	17	19	17	17	103
Social organizations	20	18	17	19	18	17	109
Total	269	266	271	266	264	270	1,606

## 4 Results

### 4.1 Quantification of stakeholders' demands for smart community development

**Figure 4:**
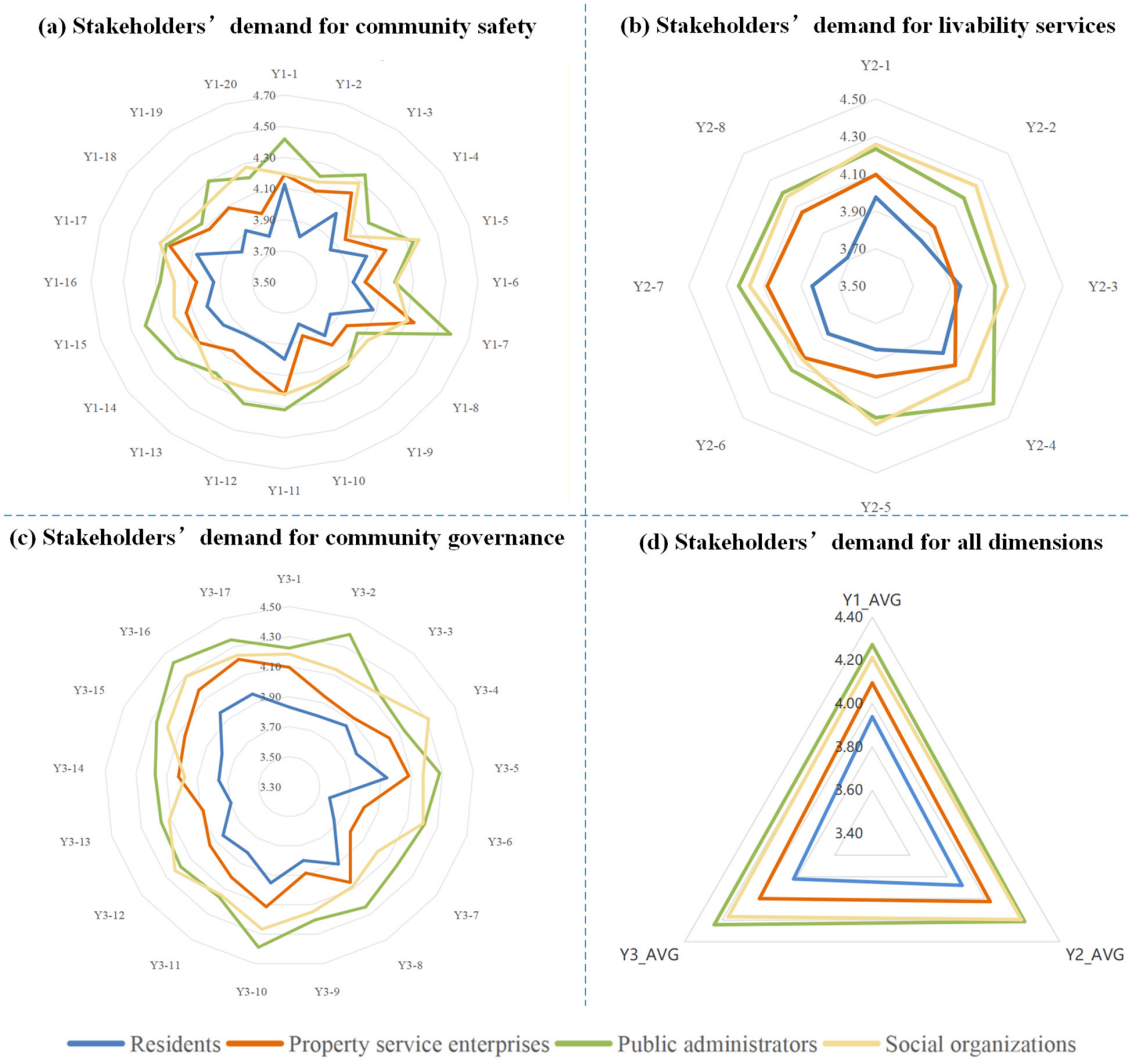
Stakeholders' demand for the smart community development. **(a)** Stakeholders' demand for community safety, **(b)** Stakeholders' demand for livability services, **(c)** Stakeholders' demand for community governance, and **(d)** Stakeholders' demand for all dimensions.

**Figure 5:**
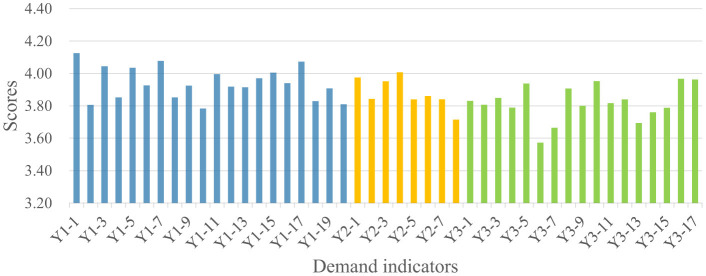
Residents' demand for the smart community development.

**Figure 6:**
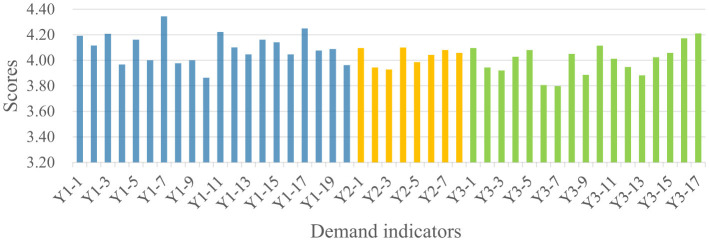
Property service enterprises' demand for the smart community development.

**Figure 7:**
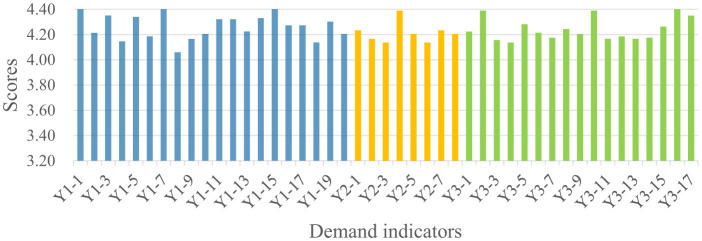
Public administrators' demand for the smart community development.

**Figure 8:**
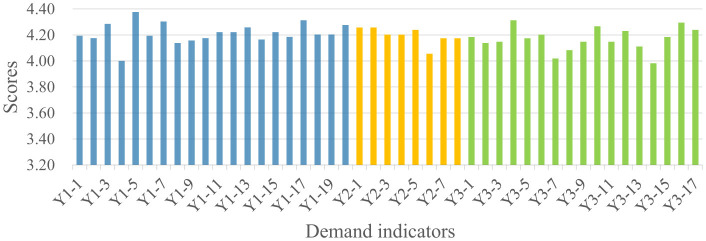
Social organizations' demand for the smart community development.

Analysis of the 1,606 valid questionnaires revealed distinct demand patterns across the four stakeholder groups, as summarized in Figure 4. Public administrators recorded the highest mean scores across all three dimensions (Y1:4.27, Y2:4.21, Y3:4.24), followed by social organizations (Y1:4.21, Y2:4.20, Y3:4.17) and property service enterprises (Y1: 4.10, Y2:4.03, Y3:4.00). Residents consistently demonstrated the lowest demand scores across all dimensions (Y1:3.94, Y2:3.88, Y3:3.82). The quantitative gaps between the highest and lowest scoring groups range from 0.33 (Community Safety) to 0.42 (Community Governance), indicating substantial heterogeneity in demand intensity across stakeholder types. Detailed indicator scores for each stakeholder group are presented in Figures 5–8.

As shown in Figure 4a, significant divergence was observed in the Community Safety dimension between public administrators (4.27) and residents (3.94). This 0.33-point gap represents an 8.4% difference relative to the scale maximum, suggesting meaningful practical divergence in safety prioritization. Detailed indicator scores indicate that public administrators scored higher than residents across all sub-indicators in this dimension. This discrepancy may reflect the greater safety pressure on public administrators, which stems from their statutory responsibilities and obligations (38). In contrast, residents' lower scores suggest that safety demands are driven more strongly by personal risk perception rather than institutional obligation. As illustrated in Figure 5, residents assigned higher scores to indicators related to personal safety, such as digital community emergency preparedness plan (Y1-1: 4.125) and life channel facilities monitoring (Y1-7: 4.077), while a lower score were assigned to public facilities monitoring (Y1-10: 3.784), an indicator related to infrastructure safety.

Regarding the Livability Services dimension (Figure 4b), the most pronounced inter-group variance was observed (score range: 3.85–4.21). Public administrators recorded the highest score (4.21), with particular emphasis on intelligent older adult care services (Y2-5; Figure 7), reflecting their prioritization of public welfare provision (39). Social organizations occupied an intermediate position (4.18), consistent with their role in advocating for service quality and coordinating welfare provision (40). Property service enterprises demonstrated notably lower score (3.99), 0.22 points below public administrators. This pattern may be attributed to market-driven incentives. Commercial entities exhibit limited interest in public service projects characterized by high investment and low returns (41), such as community health services (Y2-3; Figure 6). Residents exhibited the lowest scores (3.85), potentially attributable to constrained digital literacy and limited access to smart service platforms, which may weaken their perception of such services. As such, their demand is not fully articulated, despite underlying objective needs (42).

For the Community Governance dimension (Figure 4c), marked differentiation was evident (score range: 3.82–4.24). Public administrators (4.24) and social organizations (4.16) demonstrated elevated scores, with a narrow 0.08-point gap suggesting convergent governance priorities between institutional and civil society actors. As shown in Figures 7, 8, public administrators exhibited the strongest demand for governance efficiency improvements (43), as reflected in indicators such as collaborative community governance (Y3-2: 4.388), while social organizations prioritized social welfare enhancement, particularly support services for vulnerable groups (Y3-10: 4.312). Additionally, property service enterprises recorded moderate demand score (3.98), potentially reflecting a mismatch between their governance responsibilities and constrained administrative authority (44). Residents exhibited the lowest governance demand (3.82), with particularly low scores for community party affairs management (Y3-6: 3.572) and community volunteer management (Y3-7: 3.664). This pattern suggests that residents may prioritize governance outcomes over direct participation in governance processes (45, 46).

### 4.2 Clusters of stakeholders' demands for smart community development

**Figure 9:**
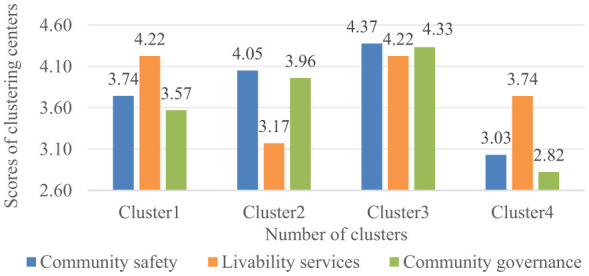
Scores of different demand clustering centers.

Inter-group analysis of demands across the three dimensions revealed distinct patterns among the four stakeholder groups (Figure 4d). To further identify underlying demand typologies, the classification model was subsequently employed. The demand classification model was employed to classify the diverse demand patterns identified in the survey data. The dataset demonstrated high reliability and validity, with a Cronbach's Alpha of 0.919 and a KMO measure of 0.961 (Bartlett's test *p* < 0.001) (47). Hierarchical clustering using Ward's method and squared Euclidean distance was first conducted, with the analysis of the agglomeration schedule indicating that the optimal solution was achieved with four clusters, beyond which the agglomeration coefficient stabilized. The agglomeration coefficient increased by 21.07% when moving from three to four clusters, but the incremental gain dropped sharply to 7.65% when moving from four to five clusters, with further declines to 6.38% (*k* = 5 to *k* = 6) and 4.39% (*k* = 6 to *k* = 7). This consistent pattern of decelerating marginal gains beyond *k* = 4 suggests that the four-cluster structure is robust against minor specification changes (a three-cluster solution would obscure meaningful distinctions, whereas a five-cluster solution would subdivide groups without theoretical justification). This four-cluster solution was subsequently validated and obtained through K-means clustering. The final cluster centers resulting from this analysis are illustrated in Figure 9.

The four clusters exhibit distinct demand profiles. Cluster 1 is characterized by a high average score for livability services, coupled with below-average scores in both community safety and governance. In contrast, Cluster 2 demonstrates a notably low demand for livability services but shows above-average demand for community safety and governance. Cluster 3 represents a comprehensive, high-demand profile, with scores exceeding the average across all three dimensions. Conversely, Cluster 4 is defined by consistently low demand intensity across community safety, livability services, and community governance.

Based on the distinct demand profiles revealed by the cluster analysis, the four clusters were designated as follows. Cluster 1 (strong livability services demand) is characterized by high demand for livability services but lower emphasis on safety and governance. Cluster 2 (prioritized safety and governance demand) shows focused demand on safety and governance dimensions with limited interest in livability services. Cluster 3 (comprehensive demand across all dimensions) represents balanced high demand across all three domains. Finally, Cluster 4 (weak livability-focused demand) demonstrates generally low demand across all dimensions with relative emphasis on basic livability services. This empirically derived classification provides a robust framework for understanding the heterogeneity in stakeholders' demands and establishes a foundation for developing tailored smart community development strategies.

## 5 Discussion

### 5.1 Evolution of stakeholders' demand clusters

**Figure 10:**
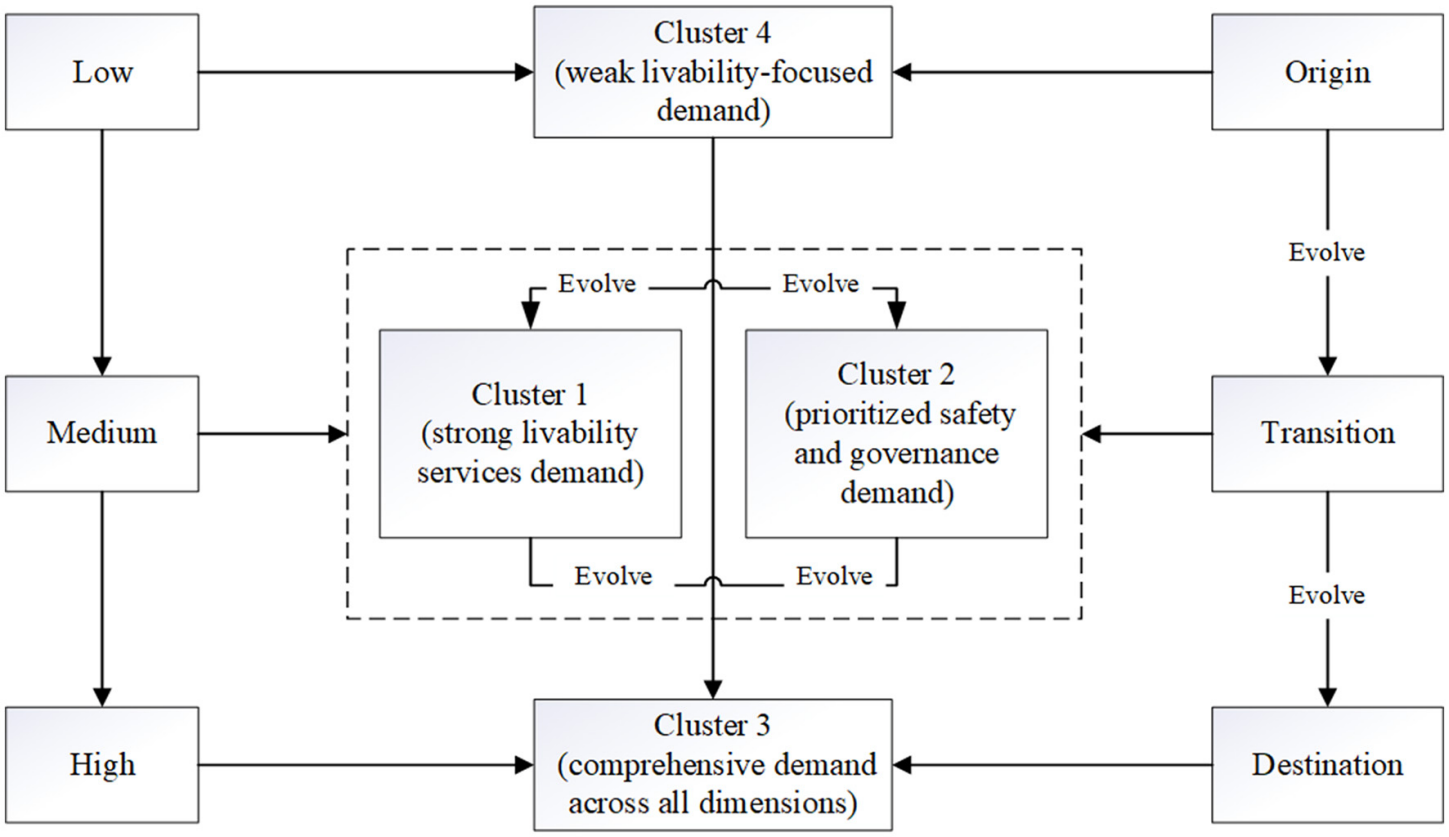
Evolution of demand clusters for smart community development.

The four identified clusters are interpreted not as static types, but as theoretically inferred stages along a developmental continuum characterized by increasing demand intensity (low → medium → high). This progression (see Figure 10) aligns with the developmental stages of smart communities, from foundational service provision to comprehensive and integrated systems (48). However, it should be emphasized that this evolutionary pathway represents a conceptual and interpretive framework, rather than an empirically observed temporal sequence, given the cross-sectional nature of the survey data. This stage-based interpretation is theoretically informed by the literature on socio-technical transitions and phased urban development, which conceptualizes urban innovation and governance transformation as gradual processes rather than abrupt shifts (49, 50). Within this perspective, smart community development is understood as a cumulative process in which community safety, livelihood services, and community governance co-evolve toward increasing integration and complexity.

The evolutionary pathway originates with Cluster 4 (weak livability-focused demand). Characterized by generally low demand across stakeholder groups, this cluster represents the initial stage of smart community development (51, 52). It is typically applicable to traditional neighborhood communities or those with limited funding, where basic service providers (e.g., property service enterprises) are introduced to establish foundational infrastructure (53). However, this stage is often hampered by governance challenges, including ill-defined responsibilities and redundant service processes, which impede further development (20, 54).

The pathway then diverges into two transitional and medium-demand stages: Cluster 1 (strong livability services demand) and Cluster 2 (prioritized safety and governance demand). Cluster 1 emerges through market mechanisms that vigorously address demands for community-based services such as healthcare and older adult care (55). A potential pitfall of this market-driven pattern is the relative neglect of public goods, notably community safety and governance (45). In contrast, Cluster 2 evolves through government mandates, prioritizing the enhancement of public safety and governance capacity. This pattern is crucial in contexts where market operations are not yet viable or business models are unclear, but its scalability is often limited by fiscal pressures and constrained service coverage (56, 57).

The destination of this evolutionary pathway is Cluster 3 (comprehensive demand across all dimensions). This cluster represents a mature, public-private partnership model where governmental bodies set standards and provide policy support, while private enterprises contribute technical expertise and manage market-oriented operations (58). Risks and benefits are clearly defined through contractual agreements, enabling a comprehensive response to the full spectrum of stakeholder demands in community safety, livability services, and community governance (59). Consequently, Cluster 3 is positioned as the most advanced and sustainable allocation for fulfilling the complex demands for the smart community.

### 5.2 Advantages of the stakeholders' demand classification model

The stakeholders' demand classification model developed in this study offers several significant advantages over previous approaches, enhancing both the theoretical understanding and practical management of smart community development.

First, the model integrates the disparate demands of all four key stakeholder groups—residents, property service enterprises, public administrators, and social organizations—into a comprehensive demand indicator system. Theoretically, this demand indicator system is grounded in Maslow's Hierarchy of Needs and the Kano Model. Maslow's Hierarchy identifies universal need categories that apply across all stakeholder groups, while the Kano Model explains how these groups differentially classify specific services as basic, performance, or excitement quality attributes. Moving beyond prior research that has typically examined stakeholder groups in isolation (60), this holistic architecture ensures that the full spectrum of community demands is captured systematically. This integration is crucial for identifying synergies and trade-offs between different stakeholders, thereby providing a more realistic and actionable foundation for policymaking (61).

Second, the classification model employs a hybrid quantitative methodology, combining hierarchical clustering with K-means clustering analysis, to objectively categorize stakeholders' demand profiles (62). This data-driven approach overcomes the constraints of purely qualitative or theoretical typologies, which may be subject to researcher bias (63, 64). The application of these clustering techniques enables the empirical identification of demand patterns directly from survey data (65), thereby establishing a reproducible protocol for classifying smart communities based on their unique demand structures.

Third, a principal theoretical contribution of this model lies in its clarification of the intrinsic relationships between different demand clusters, which are conceptualized as sequential stages in a dynamic evolutionary process (66). By introducing a “low → medium → high” demand pathway, the model moves beyond static classification to offer a developmental perspective on smart communities (67). This reveals not only where a community currently stands but also its potential trajectory, providing valuable insights into the necessary conditions for advancing to more mature and integrated phases of development.

### 5.3 Applicability of the stakeholders' demand classification model

While this study is grounded in the Chinese context, the proposed stakeholder demand taxonomy and the underlying classification model offer significant potential for cross-national application. Specifically, the three-dimensional framework—comprising community safety, livelihood services, and community governance—captures fundamental urban needs that resonate globally (25, 26, 28). Indicators such as “digital community emergency preparedness plan” (Y1-1), “community medical services” (Y2-4), and “collaborative community governance” (Y3-2) are universal components of resilient urban environments (68, 69). Furthermore, the data-driven clustering methodology (HCA and K-means) provides a robust, objective protocol for identifying demand profiles that can be replicated in any governance setting (32).

However, not all secondary indicators are equally transferable across national settings. Indicators tied to China's distinctive institutional architecture may require contextual adjustment, substitution, or redefinition to ensure conceptual equivalence. In addition, it should also be acknowledged that the empirical application of the framework is influenced by the selection of survey sites. All sampled communities in this study have been officially designated as smart community pilots, which inevitably introduces a degree of selection bias and may limit the full variability of stakeholder demand patterns. Therefore, cross-national applications of the proposed framework should not only preserve the core dimensional structure and analytical logic, but also adopt more diversified sampling strategies that include communities at different stages of smart development, while allowing for adaptive calibration of context-sensitive indicators to reflect local social, cultural, and institutional conditions.

### 5.4 Strategies to satisfy stakeholders' demands for the smart community development

Building upon the quantified demand profiles and the four distinct clusters identified, tailored strategies are proposed to effectively address the specific needs of each stakeholder group and facilitate the evolution of smart communities along the developmental pathway. These strategies move beyond a one-size-fits-all approach, advocating for targeted interventions based on a community's predominant demand characteristics.

For communities characterized by Cluster 1, policy interventions may primarily focus on promoting market innovation in high-demand service sectors. This can be achieved by establishing a regulatory environment that incentivizes private investment in domains such as community healthcare and smart care for older adults (70). Concurrently, to mitigate the risk of neglecting public goods like community safety and governance, implementing minimum service standards for smart community data security (71, 72), along with oversight mechanisms for smart community operations (73, 74), could help ensure a balanced development trajectory.

For communities falling under Cluster 2, strategic public investment is crucial to address foundational deficiencies in smart community infrastructure. Public funding could be directed toward modernizing core intelligent systems, with priority given to the establishment of centralized data hubs and integrated platforms for community safety and governance (75). To ensure the financial sustainability of these technological upgrades, innovative instruments such as municipal bonds specifically earmarked for smart city initiatives could be explored (76). Moreover, parallel administrative reforms are essential to clarify jurisdictional boundaries and streamline service workflows by implementing digital grid-based management systems. These reforms ensure that technology investments translate into tangible improvements, ultimately enhancing the efficiency and scope of government-led smart community development (77).

For communities exhibiting the comprehensive demand profile of Cluster 3, the strategic focus could shift toward establishing structured public-private partnerships that leverage the respective strengths of both sectors (78). This requires the development of standardized contractual frameworks which clearly define the responsibilities, risk-sharing arrangements, and revenue models for all involved stakeholders (79). Such legal and operational clarity is essential for mobilizing private sector investment and technical expertise in large-scale smart community projects. Subsequently, these partnerships can facilitate the coordinated delivery of integrated services, including intelligent safety systems, digital livelihood services, and data-driven governance platforms (80). Ultimately, this collaborative pattern serves as the institutional foundation for achieving balanced smart community development.

Finally, for communities identified as Cluster 4, a phased implementation strategy is recommended to establish fundamental smart community capabilities (81). Initial phases could focus on developing essential digital infrastructure, including basic connectivity and shared data platforms, while concurrently clarifying property rights and operational boundaries (82). Subsequently, professional operators can be entrusted with managing specific smart services through performance-based contracts that establish clear service-level agreements and accountability metrics (83). This structured approach addresses the current challenges of ambiguous responsibilities while building the necessary foundation for sustainable smart community development.

## 6 Conclusion

This study developed and applied a comprehensive classification model to decode the complex landscape of stakeholders' demands in smart community development. By constructing a multi-dimensional indicator system and employing a hybrid clustering methodology on a substantial dataset of 1,606 respondents from China, four distinct stakeholders' demand clusters were empirically identified: Cluster 1 (strong livability services demand), Cluster 2 (prioritized safety and governance demand), Cluster 3 (comprehensive demand), and Cluster 4 (weak, livability-focused demand). Crucially, these clusters were found not to be isolated types, but interconnected stages along a consistent low-medium-high evolutionary pathway, providing a dynamic perspective on how smart community needs mature.

The research offers dual contributions. Theoretically, it moves beyond the fragmented analysis of single stakeholders by introducing a holistic taxonomy that integrates the demands of residents, property service enterprises, public administrators, and social organizations. This paradigm shift, supported by a robust quantitative classification model, provides a new framework for understanding the dynamics of community demands. Practically, the findings deliver an actionable tool for policymakers and planners. The ability to classify a community into a specific demand profile enables the design of highly targeted development strategies, from fostering market innovation in Cluster 1 to instituting collaborative governance in Cluster 3, thereby optimizing resource allocation and enhancing the strategic impact of smart city initiatives. This evidence-based framework also moves beyond one-size-fits-all approaches by fully accommodating multi-stakeholder demands, thereby strengthening social cohesion and informing public-health preparedness and disaster-risk governance.

Despite these contributions, two limitations point to directions for future research. First, this study relies on cross-sectional data, which constrains the ability to capture the dynamic evolution of stakeholder demand structures. Future studies could employ longitudinal designs to examine how demand patterns change over time and to validate the proposed developmental pathway. Second, the empirical analysis is based on a sample of communities that have already been designated as smart community pilots, which may introduce selection bias and limit the representativeness of the findings. Although these communities exhibit heterogeneous development emphases, they may not represent the full spectrum of community development stages. Future research applying this framework to diverse international contexts and to communities at earlier development stages would strengthen its generalizability.

## Data Availability

The raw data supporting the conclusions of this article will be made available by the authors, without undue reservation.
