# Correction: Decoding stakeholders' demand to map the future of smart communities: evidence from China

**DOI:** 10.3389/fpubh.2026.1863677

**Published:** 2026-05-20

**Authors:** Wei Qi, Yujia Shan, Ling Ma, Hao Wu, Hongtu Yan, Pengju Han, Tiantian Gu

**Affiliations:** 1 School of Construction Management, Jiangsu Vocational Institute of Architectural Technology, Xuzhou, China; 2 School of Mechanics and Civil Engineering, China University of Mining and Technology, Xuzhou, China

**Keywords:** cluster analysis, community governance, community resilience, smart community, stakeholder demand

In the published article, there was an error in the first paragraph of **Section 5.2**. The first paragraph of **Section 5.2** duplicates the first paragraph of **Section 5.1**. The duplicated paragraph should be replaced with the correct original text. The duplicated text displayed as follows:

“The four identified clusters are interpreted not as static types, but as theoretically inferred stages along a developmental continuum characterized by increasing demand intensity (low → medium → high). This progression (see Figure 10) aligns with the developmental stages of smart communities, from foundational service provision to comprehensive and integrated systems (59). However, it should be emphasized that this evolutionary pathway represents a conceptual and interpretive framework, rather than an empirically observed temporal sequence, given the cross-sectional nature of the survey data. This stage-based interpretation is theoretically informed by the literature on socio-technical transitions and phased urban development, which conceptualizes urban innovation and governance transformation as gradual processes rather than abrupt shifts (60, 61). Within this perspective, smart community development is understood as a cumulative process in which community safety, livelihood services, and community governance co-evolve toward increasing integration and complexity.”

A correction has been made to the first paragraph of **Section 5.2**:

“The stakeholders' demand classification model developed in this study offers several significant advantages over previous approaches, enhancing both the theoretical understanding and practical management of smart community development.”

The original version of this article has been updated.

