# Supplementary material for: Decoding stakeholders' demand to map the future of smart communities: evidence from China
Source: Front Public Health. 2026 Mar 13;14:1751235. doi: 10.3389/fpubh.2026.1751235 (PMC13021643; doi:10.3389/fpubh.2026.1751235)
Supplement: Supplementary file 1 [file Table_1.docx]

Supplementary File S1

Table S1. The preliminary demand indicator system for smart community development

| Dimension | ID | Indicators | Residents | Property service enterprises | Public administrators | Social organizations | Sources |
| --- | --- | --- | --- | --- | --- | --- | --- |
| Community safety | L1-1 | Digital community emergency preparedness plan | √ | √ | √ | √ | References  [1-5] |
|  | L1-2 | Emergency plan implementing | √ | √ | √ | √ |  |
|  | L1-3 | Propaganda and education of emergency safety | √ | √ | √ | √ |  |
|  | L1-4 | Community staff training |  | √ | √ | √ |  |
|  | L1-5 | Abnormal events recording | √ | √ | √ | √ |  |
|  | L1-6 | Management and control of key parts | √ | √ | √ | √ |  |
|  | L1-7 | Building monitoring | √ | √ | √ | √ |  |
|  | L1-8 | Life channel facilities monitoring | √ | √ | √ | √ |  |
|  | L1-9 | Floating population services | √ | √ | √ |  |  |
|  | L1-10 | Smart object monitoring facilities | √ | √ | √ | √ |  |
|  | L1-11 | Public facilities monitoring | √ | √ | √ | √ |  |
|  | L1-12 | Smart environment monitoring | √ | √ | √ | √ |  |
|  | L1-13 | Smart firefighting facilities | √ | √ | √ | √ |  |
|  | L1-14 | Community safety inspection | √ | √ | √ | √ |  |
|  | L1-15 | Emergency duty | √ | √ | √ | √ |  |
|  | L1-16 | Intelligent emergency alert and forecasting | √ | √ | √ | √ |  |
|  | L1-17 | Coordinated emergency response | √ | √ | √ | √ |  |
|  | L1-18 | Emergency broadcast system | √ | √ | √ | √ |  |
|  | L1-19 | Emergency rescue alarm | √ | √ | √ | √ |  |
|  | L1-20 | Emergency shelter guidelines | √ | √ | √ | √ |  |
|  | L1-21 | Emergency supplies reserve | √ | √ | √ | √ |  |
|  | L1-22 | Emergency command and dispatch | √ | √ | √ | √ |  |
|  | L1-23 | Disaster risk map | √ | √ | √ | √ |  |
|  | L1-24 | Post-response community safety evaluation | √ | √ | √ | √ |  |
|  | L1-25 | Security facility management | √ | √ | √ | √ |  |
| Livability services | L2-1 | Community service center | √ | √ | √ | √ | Reference  [6-10] |
|  | L2-2 | Community self-service terminals | √ | √ | √ | √ |  |
|  | L2-3 | Community health services | √ | √ | √ |  |  |
|  | L2-4 | Community medical services | √ | √ | √ |  |  |
|  | L2-5 | Community emergency rescue services | √ | √ | √ | √ |  |
|  | L2-6 | Intelligent older adult care services | √ | √ | √ | √ |  |
|  | L2-7 | Psychological counseling services | √ | √ | √ | √ |  |
|  | L2-8 | Business management | √ | √ | √ |  |  |
|  | L2-9 | Centralized reporting & maintenance system | √ | √ | √ | √ |  |
|  | L2-10 | Feedback system for complaints and suggestions | √ | √ | √ | √ |  |
|  | L2-11 | Household waste management | √ | √ | √ | √ |  |
|  | L2-12 | Housing rental and sales system | √ | √ | √ |  |  |
|  | L2-13 | Recycling system for used things | √ | √ | √ | √ |  |
|  | L2-14 | Vehicle charging station | √ | √ | √ |  |  |
|  | L2-15 | Parcel delivery and collection system | √ | √ |  |  |  |
|  | L2-16 | **Home service reservation** | √ | √ |  |  |  |
|  | L2-17 | Digital information board | √ | √ | √ | √ |  |
|  | L2-18 | Smart childcare | √ | √ | √ | √ |  |
| Community governance | L3-1 | Community grid-based governance | √ | √ | √ | √ | References  [11-15] |
|  | L3-2 | Collaborative community governance | √ | √ | √ | √ |  |
|  | L3-3 | Social organization engagement in governance |  |  | √ | √ |  |
|  | L3-4 | Business participation in community governance |  | √ | √ |  |  |
|  | L3-5 | Community population management | √ | √ | √ | √ |  |
|  | L3-6 | Community vehicle management | √ | √ | √ | √ |  |
|  | L3-7 | Community party affairs management | √ | √ | √ | √ |  |
|  | L3-8 | Community volunteer management | √ | √ | √ | √ |  |
|  | L3-9 | Housing management | √ | √ | √ | √ |  |
|  | L3-10 | Information disclosure | √ | √ | √ | √ |  |
|  | L3-11 | Integrated government service system | √ | √ | √ | √ |  |
|  | L3-12 | Services for economically disadvantaged groups | √ | √ | √ | √ |  |
|  | L3-13 | Services for persons with disabilities | √ | √ | √ | √ |  |
|  | L3-14 | Floating population services | √ | √ | √ |  |  |
|  | L3-15 | Conflict regulation | √ | √ | √ | √ |  |
|  | L3-16 | Legal awareness and legal services | √ | √ | √ | √ |  |
|  | L3-17 | Community cultural and recreational activities | √ | √ | √ | √ |  |
|  | L3-18 | Centralized incident dispatch and monitoring | √ | √ | √ | √ |  |
|  | L3-19 | Community alert broadcasting and statistics | √ | √ | √ | √ |  |
|  | L3-20 | Multi-sectoral linkage | √ | √ | √ | √ |  |
|  | L3-21 | Monitoring of special population groups | √ | √ | √ | √ |  |
|  | L3-22 | Centralized incident dispatch and monitoring | √ | √ | √ | √ |  |

**Reference：**

1. Wang, C.Y.; Wang, L.X.; Gu, T.T.; Yin, J.Y.; Hao, E.Y. CRITIC-TOPSIS-Based Evaluation of Smart Community Safety: A Case Study of Shenzhen, China. *Buildings* **2023**, *13*, doi:10.3390/buildings13020476.

2. Sha, Y.T.; Li, M.H.; Xu, H.K.; Zhang, S.H.; Feng, T.X. Smart City Public Safety Intelligent Early Warning and Detection. *Scientific Programming* **2022**, *2022*, doi:10.1155/2022/7552601.

3. Wang, X.; Zhang, X.X.; He, J.J. Challenges to the system of reserve medical supplies for public health emergencies: reflections on the outbreak of the severe acute respiratory syndrome coronavirus 2 (SARS-CoV-2) epidemic in China. *Bioscience Trends* **2020**, *14*, 3-8, doi:10.5582/bst.2020.01043.

4. Chen, C.L.; Lim, Z.Y.; Liao, H.C. Blockchain-Based Community Safety Security System with IoT Secure Devices. *Sustainability* **2021**, *13*, doi:10.3390/su132413994.

5. Sun, J.B.; Lin, S.; Zhang, G.B.; Sun, Y.T.; Zhang, J.F.; Chen, C.F.; Morsy, A.M.; Wang, X.Y. The effect of graphite and slag on electrical and mechanical properties of electrically conductive cementitious composites. *Construction and Building Materials* **2021**, *281*, doi:10.1016/j.conbuildmat.2021.122606.

6. Barns, S.; Cosgrave, E.; Acuto, M.; McNeill, D. Digital Infrastructures and Urban Governance. *Urban Policy and Research* **2017**, *35*, 20-31, doi:10.1080/08111146.2016.1235032.

7. Tan, J.; Leng, J.; Zeng, X.D.; Feng, D.; Yu, P.L. Digital Twin for Xiegong's Architectural Archaeological Research: A Case Study of Xuanluo Hall, Sichuan, China. *Buildings* **2022**, *12*, doi:10.3390/buildings12071053.

8. Wang, F.K.; Zhang, J.Z.; Zhang, P.K. Influencing Factors of Smart Community Service Quality: Evidence from China. *Tehnicki Vjesnik-Technical Gazette* **2021**, *28*, 1187-1196, doi:10.17559/tv-20210429094941.

9. Sun, J.B.; Wang, X.Y.; Zhang, J.F.; Xiao, F.; Sun, Y.T.; Ren, Z.H.; Zhang, G.B.; Liu, S.K.; Wang, Y.F. Multi-objective optimisation of a graphite-slag conductive composite applying a BAS-SVR based model. *Journal of Building Engineering* **2021**, *44*, doi:10.1016/j.jobe.2021.103223.

10. Li, M.; Shen, J.; Wang, X.X.; Chen, Q.; Liao, X.Y.; Ren, L. A theoretical framework based on the needs of smart aged care for Chinese community-dwelling older adults: A grounded theory study. *International Journal of Nursing Knowledge* **2024**, *35*, 13-20, doi:10.1111/2047-3095.12408.

11. Gagliardi, D.; Schina, L.; Sarcinella, M.L.; Mangialardi, G.; Niglia, F.; Corallo, A. Information and communication technologies and public participation: interactive maps and value added for citizens. *Government Information Quarterly* **2017**, *34*, 153-166, doi:10.1016/j.giq.2016.09.002.

12. Ding, J.W.; Xu, J.; Weise, T.; Wang, H. Community Services and Social Involvement in COVID-19 Governance: Evidence from China. *International Journal of Environmental Research and Public Health* **2022**, *19*, doi:10.3390/ijerph192215279.

13. Guo, J.; Ling, W.H. Impact of Smart City Planning and Construction on Community Governance under Dynamic Game. *Complexity* **2021**, *2021*, doi:10.1155/2021/6690648.

14. Wan, L.J.; Jiang, S.Q. Research on the Influencing Factors of Sustainable Development of Smart Community. *Mathematical Problems in Engineering* **2022**, *2022*, doi:10.1155/2022/8420851.

15. Yin, J.Y.; Wang, J.Q.; Wang, C.Y.; Wang, L.X.; Chang, Z.Y. CRITIC-TOPSIS Based Evaluation of Smart Community Governance: A Case Study in China. *Sustainability* **2023**, *15*, doi:10.3390/su15031923.
