# Supplementary material for: Decoding stakeholders' demand to map the future of smart communities: evidence from China
Source: Front Public Health. 2026 Mar 13;14:1751235. doi: 10.3389/fpubh.2026.1751235 (PMC13021643; doi:10.3389/fpubh.2026.1751235)
Supplement: Supplementary file 2 [file Table_2.docx]

Supplementary File S2

Questionnaire on importance of demand indicators for smart community development and the corresponding results

**Supplementary File S2-1. Questionnaire on importance of demand indicators for smart community development**

Dear Sir/Madam:

Greetings! We warmly invite you to join our research initiative. Our team is currently conducting research on residents’ demands for the smart community development. To better understand your demands for smart community development, we would be most grateful for your valuable input. We hope you could take a few minutes from your busy schedule to complete this anonymous questionnaire. All responses will be kept confidential and used solely for statistical analysis. Your cooperation would be highly appreciated!

Institute of Engineering Management,

China University of Mining and Technology

**I. Basic Information**

1. What is your gender?

A. Male

B. Female

2. How many years of experience do you have in the field of smart communities?

A. Less than 1 year

B. 1–3 years

C. 4–5 years

D. More than 5 years

3. Are you from academia or industry?

A. Academia (universities or research institutions)

B. Industry (enterprises)

4. What is your professional title?

A. Doctorate

B. Master’s Degree

C. Bachelor’s Degree

D. Others (high school and below)

**II. Survey on the degree of residents’ demands for the smart community development**

Smart community construction demands are defined as the service requirements of four key stakeholders (residents, property service enterprises, administrative personnel, and social organizations) encompassing physiological, safety, social belonging, esteem, and self-actualization needs. The demand system for smart community development comprises three dimensions: community safety, livelihood services, and community governance. Specific demand items are detailed in the table below. Based on your professional expertise, please assess the importance of each demand indicator for smart community development. Please rate each item using the following scale: 1 = very unimportant, 2 = unimportant, 3 = neutral, 4 = important, and 5 = very important.

**Table S2. Survey on the importance of demand indicators for smart community development**

| Dimension | ID | Indicators | Descriptions | Importance Score |
| --- | --- | --- | --- | --- |
| Community safety | L1-1 | Digital community emergency preparedness plan | A formal emergency plan that is specifically tailored to the community’s context and made accessible in a digital format. It includes risk assessments, response protocols, and resource coordination mechanisms, facilitating efficient dissemination and stakeholder awareness. |  |
|  | L1-2 | Emergency plan implementing | Smart implementation of emergency plans (aligned with higher authorities’ requirements and community characteristics), including the establishment of community disaster/risk management institutions, risk/hazard lists, disaster response procedures, contact information, and rescue teams. |  |
|  | L1-3 | Propaganda and education of emergency safety | Dissemination of safety knowledge via radio, television, the Internet, and electronic displays. Regular safety education sessions and training on risk avoidance skills are provided for residents. |  |
|  | L1-4 | Community staff training | Provision of safety management and emergency response training for personnel of community workstations (residents’ committees) and property service enterprises. |  |
|  | L1-5 | Abnormal events recording | An abnormal events recording mechanism is established to record all kinds of accidents in the jurisdiction, including drug abuse and disputes. |  |
|  | L1-6 | Management and control of key parts | Smart information technology is used to realize information collection, monitoring, and comprehensive management of key parts such as roads, public places and violation housing in the smart community. |  |
|  | L1-7 | Building monitoring | The unmanned aerial vehicle, information sensors, big data, and other technologies are adopted to dynamically supervise the safety of building structures, and the monitoring system can realize timely warnings when there are problems in the safety of building structures. |  |
|  | L1-8 | Life channel facilities monitoring | Smart monitoring equipment is set at the life passage of the smart community, which helps to check and upload the data of fire truck passages, evacuation passages, safety exits, and other facilities in residential buildings at all times. |  |
|  | L1-9 | Floating population management | Collection and management of floating population data through intelligent visitor systems, recreational facility monitoring, smart access control, and abnormal behavior detection. |  |
|  | L1-10 | Smart object monitoring facilities | Electronic safety devices monitor community motor vehicles, high-altitude parabolic behavior, and anti-theft systems. Abnormalities trigger timely alerts to relevant personnel, helping prevent major safety accidents. |  |
|  | L1-11 | Public facilities monitoring | Intelligent information technology is used to provide information collection, registration, and residence certification services for the floating population, and provide sound cultural, physical, and health service facilities and rich service content. |  |
|  | L1-12 | Smart environment monitoring | By means of information technology, community administrators or residents can upload community environmental problems in time through mobile clients to ensure the timely disposal of community environmental problems. |  |
|  | L1-13 | Smart firefighting facilities | Smart fire-fighting facilities in the community are built, including smart fire hydrants, fire location systems, smart alarm devices, automatic firefighting devices, etc. Smart fire-fighting system is set up to realize the timely treatment of firefighting incidents. |  |
|  | L1-14 | Community safety inspection | A mechanism that utilizes digital tools (e.g., mini-programs, QR codes) to conduct systematic inspections of key community areas for security, fire safety, and sanitation. Identified issues are logged and reported through the system, with the process and outcomes made transparent to enable supervision by both residents and superior departments. |  |
|  | L1-15 | Emergency duty | The emergency workstation is set up, which is equipped with a smart emergency management system and emergency duty terminal. Relevant information is connected to the smart community emergency management duty system, and a normalized emergency team is set up to carry out community safety monitoring and early warning. |  |
|  | L1-16 | Intelligent emergency alert and forecasting | This system employs IT solutions for real-time monitoring and analysis of diverse emergency scenarios. Its objective is to generate accurate forecasts and early warnings, enabling swift incident response and effective management. |  |
|  | L1-17 | Coordinated emergency response | A multi-stakeholder system involving both relevant departments and residents, activated in response to sudden community emergencies. Its core objective is to achieve rapid containment and mitigation of hazards, thereby ensuring the safety of life and property and minimizing overall losses. |  |
|  | L1-18 | Emergency broadcast system | Extreme weather, people’s dangerous behaviors, and natural disasters in the community are monitored and warned by means of information technology to ensure that accidents can be warned and solved in time. |  |
|  | L1-19 | Emergency rescue alarm | The emergency rescue alarm system is built, and emergency help buttons and emergency communication devices with property management center and emergency control center are set up in community public areas. Emergency rescue alarm is set indoors to help community residents get in touch with medical institutions in time and push alarm information to emergency contacts. |  |
|  | L1-20 | Emergency shelter guidelines | Emergency shelter guidance services via integrated platforms and broadcast systems during incident response. |  |
|  | L1-21 | Emergency supplies reserve | By means of information technology, emergency supplies are monitored dynamically, the information on emergency supplies reserve is fed back to community administrators in time, and the information of residents is displayed on the platform so that emergency supplies reserve can be quickly gained when an emergency occurs. |  |
|  | L1-22 | Emergency command and dispatch | Community emergency service resources are integrated, including police, medical care, volunteers, and social emergency forces. The corresponding emergency rescue service dispatching mechanism is provided to community residents. |  |
|  | L1-23 | Disaster risk map | Regular hazard identification, risk assessment, and disaster risk mapping within the community. |  |
|  | L1-24 | Post-response community safety evaluation | A systematic assessment of the effectiveness of emergency measures and management following a community incident. It involves a thorough review of the emergency plan, personnel organization, equipment, and drills to generate actionable recommendations for improving emergency management capabilities. |  |
|  | L1-25 | Security facility management | Management of security facility data, encompassing video imagery, vehicle identification data, access control logs, and alarm information. |  |
| Livability services | L2-1 | Community service center | A smart community service center in accordance with urban residential infrastructure standards to provide daily life services for residents. |  |
|  | L2-2 | Community self-service terminals | A smart community terminal that consolidates functions from portal websites, community social groups, and hotlines to offer property management, payment, and commercial services to residents. |  |
|  | L2-3 | Community health services | To enhance community healthcare standards, a dedicated service system must be established. This system will ensure interconnected medical information, provide daily health management and basic monitoring, promote disease prevention, and offer free check-ups and self-diagnostics. It will also implement personal health records and a chronic disease management system. |  |
|  | L2-4 | Community medical services | Leveraging information technology to provide convenient services such as medical appointment booking for community residents. |  |
|  | L2-5 | Community emergency rescue services | Installation and maintenance of emergency equipment with rescue and first-aid capabilities. |  |
|  | L2-6 | Intelligent older adult care services | A smart community platform designed to enhance accessibility to basic retirement and health coverage for seniors. It enables seniors and their guardians to remotely access services such as weather monitoring, emergency alerts, and general support via mobile or PC applications. The platform also facilitates home-based care, including cultural and sports activities, home maintenance, shopping assistance, and medical aid. |  |
|  | L2-7 | Psychological counseling services | Referral services for psychological counseling and mental health facilities (supplementary to basic medical services). |  |
|  | L2-8 | Business management | Dynamic management of commercial establishment information through online entry and maintenance of business unit data. |  |
|  | L2-9 | Centralized reporting & maintenance system | A centralized system to streamline the reporting of community incidents and facility repairs. It provides residents with transparency into the resolution process and a direct channel for online feedback. |  |
|  | L2-10 | Feedback system for complaints and suggestions | Establishment of resident grievance and suggestion platforms for administrative appeals and policy feedback. |  |
|  | L2-11 | Household waste management | Deployment of smart waste receptacles for classification monitoring, publicity, and automated fill-level detection. |  |
|  | L2-12 | Housing rental and sales system | Dynamic housing information management covering basic property data and rental information, with resident self-service listing capabilities. |  |
|  | L2-13 | Recycling system for used things | Information technology is harnessed to build smart recycling stations that assist residents in identifying and sorting used items. Furthermore, the information about the recycled items is uploaded onto the community service platform. |  |
|  | L2-14 | Vehicle charging station | Construction of centralized electric vehicle charging infrastructure with multiple payment options and real-time parameter display. |  |
|  | L2-15 | Parcel delivery and collection system | Courier and logistics service systems utilizing mobile terminals and intelligent parcel lockers with tracking, monitoring, notification, and payment functions. |  |
|  | L2-16 | **Home service reservation** | Household service booking platforms enabling service scheduling, category selection, and online payment. |  |
|  | L2-17 | Digital information board | Installation of intelligent electronic displays (LED screens) for broadcasting, announcements, commercial queries, and public information dissemination. |  |
|  | L2-18 | Smart childcare | Community-based childcare services for infants, toddlers, and school-age children. The system includes facial recognition, behavior recording, safety alerts, emergency calls, and video monitoring (remotely accessible to authorized parents/guardians), ensuring child safety and reducing parental burdens. |  |
| Community governance | L3-1 | Community grid-based governance | This pattern establishes an interconnected ecosystem of application support, data services, and data resources. It aims to achieve timely service delivery, seamless data sharing and updating, and efficient resource management and analysis, thereby providing robust support for community governance decision-making. |  |
|  | L3-2 | Collaborative community governance | This initiative leverages smart IT to integrate systems with the 12345 citizen hotline, social organizations, and vertical regulatory agencies, ensuring seamless coordination across multiple departments and platforms. It also enables residents to participate in community deliberations, consultations, management, and oversight, as well as access relevant service information through a unified community platform. |  |
|  | L3-3 | Social organization engagement in governance | Information technology facilitation of social organization participation in community deliberation, consultation, management, and supervision. |  |
|  | L3-4 | Business participation in community governance | Information technology-enabled management and supervision of enterprise participation in community governance. |  |
|  | L3-5 | Community population management | This system utilizes smart IT to enable the dynamic collection, spatial visualization, updating, maintenance, and sharing of diverse population data. It provides services to the mobile population, including information registration and residence permit applications, and accurately collects and reports resident requests. This facilitates a virtuous cycle of interaction between citizens and the government, establishing a real-time, responsive, and sustainable management mechanism. |  |
|  | L3-6 | Community vehicle management | This system utilizes smart information technology to enable the dynamic collection, spatial visualization, updating, maintenance, and sharing of data for all vehicles entering the community. |  |
|  | L3-7 | Community party affairs management | This platform enables the community to disseminate Party activity information and collect feedback, release announcements from Party organizations, and solicit opinions and suggestions from Party members. |  |
|  | L3-8 | Community volunteer management | This system utilizes smart information technology to handle the registration, updating, and maintenance of community volunteer information, enabling comprehensive and streamlined management. |  |
|  | L3-9 | Housing management | Intelligent information technology is adopted to realize the dynamic collection, update, maintenance, reporting, and management of housing-related information in the com-munity, including basic housing information, housing rental information, and housing safety status information. |  |
|  | L3-10 | Information disclosure | Timely disclosure of community governance information and public policies affecting residents' daily lives. |  |
|  | L3-11 | Integrated government service system | This system leverages smart information technology to extend public services for individual citizens to the community level. Through online platforms, self-service kiosks, or service counters, it provides residents with convenient, high-quality, and efficient ‘one-stop’ services. |  |
|  | L3-12 | Services for economically disadvantaged groups | Dynamic management and support services for economically disadvantaged residents. The system manages registration for aid, identifies economic assistance targets, provides information on social security and pension schemes, and offers other essential support services. |  |
|  | L3-13 | Support services for vulnerable groups | Dynamic management and support services for vulnerable groups, covering employment, social security, rehabilitation, education, and accessibility. |  |
|  | L3-14 | Floating population services | Services for the floating population, including information collection, registration, residence permits, and access to cultural, sports, and health facilities. |  |
|  | L3-15 | Conflict regulation | Technology-enabled community dispute mediation with documentation and real-time reporting capabilities. |  |
|  | L3-16 | Legal awareness and legal services | Establishment of integrated legal service platforms consolidating lawyers, notaries, legal aid providers, and volunteers for community legal services. |  |
|  | L3-17 | Community cultural and recreational activities | This initiative leverages digital channels to facilitate a variety of activities planned, initiated, and organized by community staff. It aims to enrich the lives of residents, promote cultural enrichment, and foster community harmony. |  |
|  | L3-18 | Centralized incident dispatch and monitoring | This process involves dispatching incidents to relevant departments or personnel based on their nature and urgency. The entire handling process is monitored in real-time, with progress tracked and recorded to ensure proper management at every stage and to facilitate timely coordination for issue resolution, thereby enhancing the efficiency and quality of incident management. |  |
|  | L3-19 | Community alert broadcasting and statistics | This system provides real-time alert services via smart terminals, enabling the broadcasting of targeted alerts to residents in affected areas based on disaster severity levels. It also compiles statistics on emergency warnings to effectively evaluate the dissemination of critical incident information. |  |
|  | L3-20 | Multi-sectoral linkage | With the combination of online and offline, a multi-sectoral linkage mechanism of the smart community (Ministry of Housing and Urban Rural Development, Fire Department, Ministry of Public Security, etc.) is established to realize the rapid handling of emergencies. |  |
|  | L3-21 | Monitoring of special population groups | Refined management and services for key community groups (e.g., individuals with a history of public disturbances, mental health conditions, released prisoners, those under community corrections, drug users, juvenile offenders). Detailed records of basic information and activities are maintained to enhance digital management. |  |
|  | L3-22 | Information management for vulnerable groups | Digital information management and safety monitoring for vulnerable populations (including children, women, older adults, persons with disabilities, and persons with mental health conditions), encompassing the maintenance of basic information, care record updates, and location-enabled emergency response support to safeguard fundamental rights and welfare. |  |

**Ⅲ. Expert Validation of Dimensional Classification**

1. To what extent do you agree that the following indicators are appropriately classified under Community Safety? Please indicate any indicators you believe should be reassigned to Livability Services or Community Governance, and briefly explain your reasoning.
2. To what extent do you agree that the following indicators are appropriately classified under Livability Services? Please indicate any indicators you believe should be reassigned to Community Safety or Community Governance, and briefly explain your reasoning.
3. To what extent do you agree that the following indicators are appropriately classified under Community Governance? Please indicate any indicators you believe should be reassigned to Community Safety or Livability Services, and briefly explain your reasoning.

**Ⅳ. Your other suggestions for the smart community development:**

---------------------------------------------------------------------------------------

**Thanks!**

**Supplementary File S2-2.** **The questionnaire results**

1. **Basic Information**

**Table S3. Basic information of the 38 experts surveyed**

| **Variable** | **Items** | **Percentage (%)** |
| --- | --- | --- |
| Gender | Male | 47.37 |
|  | Female | 52.63 |
| Years of Experience | Over 5 years | 18.42 |
|  | 4-5 years | 31.58 |
|  | 1-3 years | 23.68 |
|  | Within one year | 26.32 |
| Professional Field | Academia | 39.47 |
|  | Enterprise | 60.53 |
| Professional title | Doctorate | 31.58 |
|  | Master’s Degree | 44.74 |
|  | Bachelor’s Degree | 18.42 |
|  | Others (high school and below) | 5.26 |

**II. Membership Degree Survey Results of Stakeholder Demand Indicators for Smart Community Development**

**Table S4. Membership Degree Survey Results**

| Dimension | ID | Indicators | Membership degree |
| --- | --- | --- | --- |
| Community safety | L1-1 | Digital community emergency preparedness plan | 0.75 |
|  | L1-2 | Emergency plan implementing | 0.75 |
|  | L1-3 | Propaganda and education of emergency safety | 0.83 |
|  | L1-4 | Community staff training | 0.64 |
|  | L1-5 | Abnormal events recording | 0.75 |
|  | L1-6 | Management and control of key parts | 0.74 |
|  | L1-7 | Building monitoring | 0.74 |
|  | L1-8 | Life channel facilities monitoring | 0.79 |
|  | L1-9 | Floating population management | 0.83 |
|  | L1-10 | Smart object monitoring facilities | 0.70 |
|  | L1-11 | Public facilities monitoring | 0.67 |
|  | L1-12 | Smart environment monitoring | 0.71 |
|  | L1-13 | Smart firefighting facilities | 0.75 |
|  | L1-14 | Community safety inspection | 0.70 |
|  | L1-15 | Emergency duty | 0.79 |
|  | L1-16 | Intelligent emergency alert and forecasting | 0.75 |
|  | L1-17 | Coordinated emergency response | 0.76 |
|  | L1-18 | Emergency broadcast system | 0.75 |
|  | L1-19 | Emergency rescue alarm | 0.79 |
|  | L1-20 | Emergency shelter guidelines | 0.49 |
|  | L1-21 | Emergency supplies reserve | 0.79 |
|  | L1-22 | Emergency command and dispatch | 0.76 |
|  | L1-23 | Disaster risk map | 0.49 |
|  | L1-24 | Post-response community safety evaluation | 0.67 |
|  | L1-25 | Security facility management | 0.34 |
| Livability services | L2-1 | Community service center | 0.76 |
|  | L2-2 | Community self-service terminals | 0.71 |
|  | L2-3 | Community health services | 0.75 |
|  | L2-4 | Community medical services | 0.72 |
|  | L2-5 | Community emergency rescue services | 0.74 |
|  | L2-6 | Intelligent older adult care services | 0.74 |
|  | L2-7 | Psychological counseling services | 0.49 |
|  | L2-8 | Business management | 0.47 |
|  | L2-9 | Centralized reporting & maintenance system | 0.75 |
|  | L2-10 | Feedback system for complaints and suggestions | 0.43 |
|  | L2-11 | Household waste management | 0.76 |
|  | L2-12 | Housing rental and sales system | 0.32 |
|  | L2-13 | Recycling system for used things | 0.42 |
|  | L2-14 | Vehicle charging station | 0.43 |
|  | L2-15 | Parcel delivery and collection system | 0.45 |
|  | L2-16 | **Home service reservation** | 0.33 |
|  | L2-17 | Digital information board | 0.33 |
|  | L2-18 | Smart childcare | 0.72 |
| Community governance | L3-1 | Community grid-based governance | 0.76 |
|  | L3-2 | Collaborative community governance | 0.75 |
|  | L3-3 | Social organization engagement in governance | 0.41 |
|  | L3-4 | Business participation in community governance | 0.49 |
|  | L3-5 | Community population management | 0.79 |
|  | L3-6 | Community vehicle management | 0.76 |
|  | L3-7 | Community party affairs management | 0.75 |
|  | L3-8 | Community volunteer management | 0.75 |
|  | L3-9 | Housing management | 0.72 |
|  | L3-10 | Information disclosure | 0.26 |
|  | L3-11 | Integrated government service system | 0.75 |
|  | L3-12 | Services for economically disadvantaged groups | 0.74 |
|  | L3-13 | Support services for vulnerable groups | 0.78 |
|  | L3-14 | Floating population services | 0.78 |
|  | L3-15 | Conflict regulation | 0.71 |
|  | L3-16 | Legal awareness and legal services | 0.79 |
|  | L3-17 | Community cultural and recreational activities | 0.76 |
|  | L3-18 | Centralized incident dispatch and monitoring | 0.84 |
|  | L3-19 | Community alert broadcasting and statistics | 0.78 |
|  | L3-20 | Multi-sectoral linkage | 0.80 |
|  | L3-21 | Monitoring of special population groups | 0.78 |
|  | L3-22 | Information management for vulnerable groups | 0.79 |

**Ⅲ. Results of Expert Validation of Dimensional Classification.**

A total of 38 experts participated in the validation exercise for external validity of the indicator system. Of these, 34 experts (89.5%) fully endorsed the proposed dimensional structure without modification, indicating strong consensus regarding the conceptual boundaries and operational definitions of the three primary dimensions. Four experts (10.5%) provided specific recommendations for indicator reclassification or consolidation to enhance discriminant validity and reduce conceptual overlap.

Based on expert recommendations regarding conceptual overlap, the indicator framework was refined. Three indicators were reassigned to alternative dimensions. ‘Smart environment monitoring’ was reassigned to Community Governance, ‘Community emergency rescue services’ was reclassified to Community Safety, and ‘Feedback system for complaints and suggestions’ was relocated to Community Governance. Moreover, three indicator pairs were consolidated to enhance discriminant validity and operational clarity. ‘Conflict regulation’ and ‘Legal awareness and legal services’ were merged into ‘Dispute mediation and legal outreach’. ‘Floating population services’ and ‘Floating population monitoring’ were combined into ‘Floating population management’. ‘Community emergency rescue services’ (following its reclassification to Community Safety) and ‘Emergency rescue alarm’ were integrated into ‘Emergency rescue alarm’.

Following these adjustments, the final demand indicator system comprises three primary dimensions and 45 secondary indicators, specifically including 20 indicators for Community Safety, 8 indicators for Livability Services, and 17 indicators for Community Governance. The high rate of expert consensus (89.5% complete agreement) and the targeted refinements based on minority expert input collectively support the external validity of the indicator system, demonstrating that the dimensional structure is robust, conceptually coherent, and applicable across diverse smart community contexts.

**Ⅳ. Other suggestions for the smart community development.**

NONE.
