# Supplementary material for: Decoding stakeholders' demand to map the future of smart communities: evidence from China
Source: Front Public Health. 2026 Mar 13;14:1751235. doi: 10.3389/fpubh.2026.1751235 (PMC13021643; doi:10.3389/fpubh.2026.1751235)
Supplement: Supplementary file 3 [file Table_3.docx]

Supplementary File S3

Table S5. The final demand indicator system for smart community development

| Dimension | ID | Indicators | Descriptions | Sources |
| --- | --- | --- | --- | --- |
| Community Safety | Y1-1 | Digital community emergency preparedness plan | A formal emergency plan that is specifically tailored to the community's context and made accessible in a digital format. It includes risk assessments, response protocols, and resource coordination mechanisms, facilitating efficient dissemination and stakeholder awareness. | References(1-5) |
|  | Y1-2 | Emergency plan implementing | Smart implementation of emergency plans (aligned with higher authorities’ requirements and community characteristics), including the establishment of community disaster/risk management institutions, risk/hazard lists, disaster response procedures, contact information, and rescue teams. |  |
|  | Y1-3 | Propaganda and education of emergency safety | Dissemination of safety knowledge via radio, television, the Internet, and electronic displays. Regular safety education sessions and training on risk avoidance skills are provided for residents. |  |
|  | Y1-4 | Abnormal events recording | An abnormal events recording mechanism is established to record all kinds of accidents in the jurisdiction, including drug abuse and disputes. |  |
|  | Y1-5 | Management and control of key parts | Smart information technology is used to realize information collection, monitoring, and comprehensive management of key parts such as roads, public places and violation housing in the smart community. |  |
|  | Y1-6 | Building monitoring | The unmanned aerial vehicle, information sensors, big data, and other technologies are adopted to dynamically supervise the safety of building structures, and the monitoring system can realize timely warnings when there are problems in the safety of building structures. |  |
|  | Y1-7 | Life channel facilities monitoring | Smart monitoring equipment is set at the life passage of the smart community, which helps to check and upload the data of fire truck passages, evacuation passages, safety exits, and other facilities in residential buildings at all times. |  |
|  | Y1-8 | Floating population management | Intelligent information technology is used to provide information collection, registration, and residence certification services for the floating population, and provide sound cultural, physical, and health service facilities and rich service content. |  |
|  | Y1-9 | Smart object monitoring facilities | Electronic safety devices monitor community motor vehicles, high-altitude parabolic behavior, and anti-theft systems. Abnormalities trigger timely alerts to relevant personnel, helping prevent major safety accidents. |  |
|  | Y1-10 | Public facilities monitoring | The running state of some public facilities is smartly monitored by the Internet of Things technology, and an alarm is generated in time when an abnormality is found. |  |
|  | Y1-11 | Smart firefighting facilities | Smart fire-fighting facilities in the community are built, including smart fire hydrants, fire location systems, smart alarm devices, automatic firefighting devices, etc. Smart fire-fighting system is set up to realize the timely treatment of firefighting incidents. |  |
|  | Y1-12 | Community safety inspection | A mechanism that utilizes digital tools (e.g., mini-programs, QR codes) to conduct systematic inspections of key community areas for security, fire safety, and sanitation. Identified issues are logged and reported through the system, with the process and outcomes made transparent to enable supervision by both residents and superior departments. |  |
|  | Y1-13 | Emergency duty | The emergency workstation is set up, which is equipped with a smart emergency management system and emergency duty terminal. Relevant information is connected to the smart community emergency management duty system, and a normalized emergency team is set up to carry out community safety monitoring and early warning. |  |
|  | Y1-14 | Intelligent emergency alert and forecasting | This system employs IT solutions for real-time monitoring and analysis of diverse emergency scenarios. Its objective is to generate accurate forecasts and early warnings, enabling swift incident response and effective management. |  |
|  | Y1-15 | Coordinated emergency response | A multi-stakeholder system involving both relevant departments and residents, activated in response to sudden community emergencies. Its core objective is to achieve rapid containment and mitigation of hazards, thereby ensuring the safety of life and property and minimizing overall losses. |  |
|  | Y1-16 | Emergency broadcast system | Extreme weather, people's dangerous behaviors, and natural disasters in the community are monitored and warned by means of information technology to ensure that accidents can be warned and solved in time. |  |
|  | Y1-17 | Emergency rescue alarm | The emergency rescue alarm system is built, and emergency help buttons and emergency communication devices with property management center and emergency control center are set up in community public areas. Emergency rescue alarm is set indoors to help community residents get in touch with medical institutions in time and push alarm information to emergency contacts. |  |
|  | Y1-18 | Emergency supplies reserve | By means of information technology, emergency supplies are monitored dynamically, the information on emergency supplies reserve is fed back to community administrators in time, and the information of residents is displayed on the platform so that emergency supplies reserve can be quickly gained when an emergency occurs. |  |
|  | Y1-19 | Emergency command and dispatch | Community emergency service resources are integrated, including police, medical care, volunteers, and social emergency forces. The corresponding emergency rescue service dispatching mechanism is provided to community residents. |  |
|  | Y1-20 | Post-response community safety evaluation | A systematic assessment of the effectiveness of emergency measures and management following a community incident. It involves a thorough review of the emergency plan, personnel organization, equipment, and drills to generate actionable recommendations for improving emergency management capabilities. |  |
| Livability Services | Y2-1 | Community service center | A smart community service center in accordance with urban residential infrastructure standards to provide daily life services for residents. | Reference(6-10) |
|  | Y2-2 | Community self-service terminals | A smart community terminal that consolidates functions from portal websites, community social groups, and hotlines to offer property management, payment, and commercial services to residents. |  |
|  | Y2-3 | Community health services | To enhance community healthcare standards, a dedicated service system must be established. This system will ensure interconnected medical information, provide daily health management and basic monitoring, promote disease prevention, and offer free check-ups and self-diagnostics. It will also implement personal health records and a chronic disease management system. |  |
|  | Y2-4 | Community medical services | Leveraging information technology to provide convenient services such as medical appointment booking for community residents. |  |
|  | Y2-5 | Intelligent older adult care services | A smart community platform designed to enhance accessibility to basic retirement and health coverage for seniors. It enables seniors and their guardians to remotely access services such as weather monitoring, emergency alerts, and general support via mobile or PC applications. The platform also facilitates home-based care, including cultural and sports activities, home maintenance, shopping assistance, and medical aid. |  |
|  | Y2-6 | Centralized reporting & maintenance system | A centralized system to streamline the reporting of community incidents and facility repairs. It provides residents with transparency into the resolution process and a direct channel for online feedback. |  |
|  | Y2-7 | Recycling system for used things | A smart recycling station supported by information technology. It can assist residents in identifying and sorting recyclables. Data on recycled materials is uploaded to the community service platform. |  |
|  | Y2-8 | Smart childcare | Community-based childcare services for infants, toddlers, and school-age children. The system includes facial recognition, behavior recording, safety alerts, emergency calls, and video monitoring (remotely accessible to authorized parents/guardians), ensuring child safety and reducing parental burdens. |  |
| Community Governance | Y3-1 | Community grid-based governance | This pattern establishes an interconnected ecosystem of application support, data services, and data resources. It aims to achieve timely service delivery, seamless data sharing and updating, and efficient resource management and analysis, thereby providing robust support for community governance decision-making. | References(11-15) |
|  | Y3-2 | Collaborative community governance | This initiative leverages smart IT to integrate systems with the 12345 citizen hotline, social organizations, and vertical regulatory agencies, ensuring seamless coordination across multiple departments and platforms. It also enables residents to participate in community deliberations, consultations, management, and oversight, as well as access relevant service information through a unified community platform. |  |
|  | Y3-3 | Smart environment monitoring | By means of information technology, community administrators or residents can upload community environmental problems in time through mobile clients to ensure the timely disposal of community environmental problems. |  |
|  | Y3-4 | Community population management | This system utilizes smart IT to enable the dynamic collection, spatial visualization, updating, maintenance, and sharing of diverse population data. It provides services to the mobile population, including information registration and residence permit applications, and accurately collects and reports resident requests. This facilitates a virtuous cycle of interaction between citizens and the government, establishing a real-time, responsive, and sustainable management mechanism. |  |
|  | Y3-5 | Community vehicle management | This system utilizes smart information technology to enable the dynamic collection, spatial visualization, updating, maintenance, and sharing of data for all vehicles entering the community. |  |
|  | Y3-6 | Community party affairs management | This platform enables the community to disseminate Party activity information and collect feedback, release announcements from Party organizations, and solicit opinions and suggestions from Party members. |  |
|  | Y3-7 | Community volunteer management | This system utilizes smart information technology to handle the registration, updating, and maintenance of community volunteer information, enabling comprehensive and streamlined management. |  |
|  | Y3-8 | Housing management | Intelligent information technology is adopted to realize the dynamic collection, update, maintenance, reporting, and management of housing-related information in the com-munity, including basic housing information, housing rental information, and housing safety status information. |  |
|  | Y3-9 | Integrated government service system | This system leverages smart information technology to extend public services for individual citizens to the community level. Through online platforms, self-service kiosks, or service counters, it provides residents with convenient, high-quality, and efficient ‘one-stop’ services. |  |
|  | Y3-10 | Support services for vulnerable groups | Digital administration of social assistance for economically disadvantaged groups, encompassing the digital registration of aid applications, identification of assistance beneficiaries, dissemination of social security and pension scheme information, and coordination of essential support services. |  |
|  | Y3-11 | Dispute mediation and legal outreach (conflict regulation) | Intelligent information technology is used to carry out community conflict mediation, which realizes the recording and real-time reporting of event information in the process of mediation and realizes the step-by-step process management of conflict registration, classified acceptance, mediation processing, and reply filing. |  |
|  | Y3-12 | Community cultural and recreational activities | This initiative leverages digital channels to facilitate a variety of activities planned, initiated, and organized by community staff. It aims to enrich the lives of residents, promote cultural enrichment, and foster community harmony. |  |
|  | Y3-13 | Centralized incident dispatch and monitoring | This process involves dispatching incidents to relevant departments or personnel based on their nature and urgency. The entire handling process is monitored in real-time, with progress tracked and recorded to ensure proper management at every stage and to facilitate timely coordination for issue resolution, thereby enhancing the efficiency and quality of incident management. |  |
|  | Y3-14 | Community alert broadcasting and statistics | This system provides real-time alert services via smart terminals, enabling the broadcasting of targeted alerts to residents in affected areas based on disaster severity levels. It also compiles statistics on emergency warnings to effectively evaluate the dissemination of critical incident information. |  |
|  | Y3-15 | Multi-sectoral linkage | With the combination of online and offline, a multi-sectoral linkage mechanism of the smart community (Ministry of Housing and Urban Rural Development, Fire Department, Ministry of Public Security, etc.) is established to realize the  rapid handling of emergencies. |  |
|  | Y3-16 | Monitoring of special population groups | Refined management and services for key community groups (e.g., individuals with a history of public disturbances, mental health conditions, released prisoners, those under community corrections, drug users, juvenile offenders). Detailed records of basic information and activities are maintained to enhance digital management. |  |
|  | Y3-17 | Information management for vulnerable groups | Digital information management and safety monitoring for vulnerable populations (including children, women, older adults, persons with disabilities, and persons with mental health conditions), encompassing the maintenance of basic information, care record updates, and location-enabled emergency response support to safeguard fundamental rights and welfare. |  |

**Reference:**

1. Wang CY, Wang LX, Gu TT, Yin JY, Hao EY. CRITIC-TOPSIS-Based Evaluation of Smart Community Safety: A Case Study of Shenzhen, China. *Buildings*. (2023) 13. doi:10.3390/buildings13020476.

2. Sha YT, Li MH, Xu HK, Zhang SH, Feng TX. Smart City Public Safety Intelligent Early Warning and Detection. *Scientific Programming*. (2022) 2022. doi:10.1155/2022/7552601.

3. Wang X, Zhang XX, He JJ. Challenges to the system of reserve medical supplies for public health emergencies: reflections on the outbreak of the severe acute respiratory syndrome coronavirus 2 (SARS-CoV-2) epidemic in China. *Bioscience Trends*. (2020) 14:3-8. doi:10.5582/bst.2020.01043.

4. Chen CL, Lim ZY, Liao HC. Blockchain-Based Community Safety Security System with IoT Secure Devices. *Sustainability*. (2021) 13. doi:10.3390/su132413994.

5. Sun JB, Lin S, Zhang GB, Sun YT, Zhang JF, Chen CF, et al. The effect of graphite and slag on electrical and mechanical properties of electrically conductive cementitious composites. *Construction and Building Materials*. (2021) 281. doi:10.1016/j.conbuildmat.2021.122606.

6. Barns S, Cosgrave E, Acuto M, McNeill D. Digital Infrastructures and Urban Governance. *Urban Policy and Research*. (2017) 35:20-31. doi:10.1080/08111146.2016.1235032.

7. Tan J, Leng J, Zeng XD, Feng D, Yu PL. Digital Twin for Xiegong's Architectural Archaeological Research: A Case Study of Xuanluo Hall, Sichuan, China. *Buildings*. (2022) 12. doi:10.3390/buildings12071053.

8. Wang FK, Zhang JZ, Zhang PK. Influencing Factors of Smart Community Service Quality: Evidence from China. *Tehnicki Vjesnik-Technical Gazette*. (2021) 28:1187-96. doi:10.17559/tv-20210429094941.

9. Sun JB, Wang XY, Zhang JF, Xiao F, Sun YT, Ren ZH, et al. Multi-objective optimisation of a graphite-slag conductive composite applying a BAS-SVR based model. *Journal of Building Engineering*. (2021) 44. doi:10.1016/j.jobe.2021.103223.

10. Li M, Shen J, Wang XX, Chen Q, Liao XY, Ren L. A theoretical framework based on the needs of smart aged care for Chinese community-dwelling older adults: A grounded theory study. *International Journal of Nursing Knowledge*. (2024) 35:13-20. doi:10.1111/2047-3095.12408.

11. Gagliardi D, Schina L, Sarcinella ML, Mangialardi G, Niglia F, Corallo A. Information and communication technologies and public participation: interactive maps and value added for citizens. *Government Information Quarterly*. (2017) 34:153-66. doi:10.1016/j.giq.2016.09.002.

12. Ding JW, Xu J, Weise T, Wang H. Community Services and Social Involvement in COVID-19 Governance: Evidence from China. *International Journal of Environmental Research and Public Health*. (2022) 19. doi:10.3390/ijerph192215279.

13. Guo J, Ling WH. Impact of Smart City Planning and Construction on Community Governance under Dynamic Game. *Complexity*. (2021) 2021. doi:10.1155/2021/6690648.

14. Wan LJ, Jiang SQ. Research on the Influencing Factors of Sustainable Development of Smart Community. *Mathematical Problems in Engineering*. (2022) 2022. doi:10.1155/2022/8420851.

15. Yin JY, Wang JQ, Wang CY, Wang LX, Chang ZY. CRITIC-TOPSIS Based Evaluation of Smart Community Governance: A Case Study in China. *Sustainability*. (2023) 15. doi:10.3390/su15031923.
