# Supplementary material for: Decoding stakeholders' demand to map the future of smart communities: evidence from China
Source: Front Public Health. 2026 Mar 13;14:1751235. doi: 10.3389/fpubh.2026.1751235 (PMC13021643; doi:10.3389/fpubh.2026.1751235)
Supplement: Supplementary file 4 [file Table_4.docx]

Supplementary File S4

Questionnaires on Stakeholders’ Demands for Smart Community Development.

**Supplementary File S4-1. Questionnaire on residents’ demands for smart community development**

Dear Sir/Madam:

Greetings! We warmly invite you to join our research initiative. Our team is currently conducting research on residents’ demands for the smart community development. To better understand your demands for smart community development, we would be most grateful for your valuable input. We hope you could take a few minutes from your busy schedule to complete this anonymous questionnaire. All responses will be kept confidential and used solely for statistical analysis. Your cooperation would be highly appreciated!

Institute of Engineering Management,

China University of Mining and Technology

**I. Basic Information**

1. What is your gender?

A. Male

B. Female

2. What is your age?

A. 17 or younger

B. 18-25 years old

C. 26-30 years old

D. 31-40 years old

E. 41-49 years old

F. 50 or older

3. What is your highest level of education completed?

A. Primary school or below

B. Junior high school

C. High school or secondary specialized school

D. Associate degree

E. Bachelor's degree

F. Postgraduate degree or higher

4. How long have you lived in your current community?

A. Less than 1 year

B. 1 to 3 years

C. More than 3 years

5. What is your average monthly disposable income? (Includes wage income, business income, property income, and transfer income)

A. Under ¥3,000

B. ¥3,000 - ¥4,999

C. ¥5,000 - ¥6,999

D. ¥7,000 - ¥8,999

E. Over ¥9,000

6. How familiar are you with smart community services?

A. Very familiar

B. Familiar

C. Neutral / Somewhat familiar

D. Unfamiliar

E. Very unfamiliar

**II. Survey on the degree of residents’ demands for the smart community development**

The resident demand system for smart community development comprises three categories: community safety, livability services, and community governance. Based on your personal living experience, please indicate your need for the following services in your community. How would you rate your need for each service? (Please use the scale: 1 = strongly unnecessary, 2 = unnecessary, 3 = neutral, 4 = necessary, and 5 = strongly necessary)

**Table S1. Survey on the Degree of Residents’ Demands for Smart Community Development**

| Dimension | ID | Indicators | Score |
| --- | --- | --- | --- |
| Community Safety | Y1-1 | Digital community emergency preparedness plan |  |
|  | Y1-2 | Emergency plan implementing |  |
|  | Y1-3 | Propaganda and education of emergency safety |  |
|  | Y1-4 | Abnormal events recording |  |
|  | Y1-5 | Management and control of key parts |  |
|  | Y1-6 | Building monitoring |  |
|  | Y1-7 | Life channel facilities monitoring |  |
|  | Y1-8 | Floating population services |  |
|  | Y1-9 | Smart object monitoring facilities |  |
|  | Y1-10 | Public facilities monitoring |  |
|  | Y1-11 | Smart firefighting facilities |  |
|  | Y1-12 | Community safety inspection |  |
|  | Y1-13 | Emergency duty |  |
|  | Y1-14 | Intelligent emergency alert and forecasting |  |
|  | Y1-15 | Coordinated emergency response |  |
|  | Y1-16 | Emergency broadcast system |  |
|  | Y1-17 | Emergency rescue alarm |  |
|  | Y1-18 | Emergency supplies reserve |  |
|  | Y1-19 | Emergency command and dispatch |  |
|  | Y1-20 | Post-response community safety evaluation |  |
| Livability Services | Y2-1 | Community service center |  |
|  | Y2-2 | Community self-service terminals |  |
|  | Y2-3 | Community health services |  |
|  | Y2-4 | Community medical services |  |
|  | Y2-5 | Intelligent older adult care services |  |
|  | Y2-6 | Centralized reporting & maintenance system |  |
|  | Y2-7 | Recycling system for used things |  |
|  | Y2-8 | Smart childcare |  |
| Community Governance | Y3-1 | Community grid-based governance |  |
|  | Y3-2 | Collaborative community governance |  |
|  | Y3-3 | Smart environment monitoring |  |
|  | Y3-4 | Community population management |  |
|  | Y3-5 | Community vehicle management |  |
|  | Y3-6 | Community party affairs management |  |
|  | Y3-7 | Community volunteer management |  |
|  | Y3-8 | Housing management |  |
|  | Y3-9 | Integrated government service system |  |
|  | Y3-10 | Support services for vulnerable groups |  |
|  | Y3-11 | Dispute mediation and legal outreach (conflict regulation) |  |
|  | Y3-12 | Community cultural and recreational activities |  |
|  | Y3-13 | Centralized incident dispatch and monitoring |  |
|  | Y3-14 | Community alert broadcasting and statistics |  |
|  | Y3-15 | Multi-sectoral linkage |  |
|  | Y3-16 | Monitoring of special population groups |  |
|  | Y3-17 | Support for vulnerable groups |  |

Ⅲ. Your other suggestions for the smart community development:

**Supplementary File S4-2. Questionnaire on** **property service enterprises’ demands for smart community development**

Dear Sir/Madam:

Greetings! We warmly invite you to join our research initiative. Our team is currently conducting research on property service enterprises’ demands for the smart community development. To better understand your demands for smart community development, we would be most grateful for your valuable input. We hope you could take a few minutes from your busy schedule to complete this anonymous questionnaire. All responses will be kept confidential and used solely for statistical analysis. Your cooperation would be highly appreciated!

Institute of Engineering Management,

China University of Mining and Technology

**I. Basic Information**

1. How many households does your property management company serve?

A. 1,000 households or fewer

B. 1,000–1,999 households

C. 2,000–2,999 households

D. 3,000–3,999 households

E. 4,000 households or more

2. How many employees does your property management company have in this community?

A. 20 or fewer

B. 21–40 employees

C. 41–60 employees

D. 61–80 employees

E. 81–100 employees

F. More than 100 employees

3. How many years of experience do you have in property services?

A. 1 year or less

B. 1–3 years

C. 4–5 years

D. More than 5 years

4. What is your average monthly income (in RMB)?

A. Under ¥3,000

B. ¥3,000 - ¥4,999

C. ¥5,000 - ¥6,999

D. ¥7,000 - ¥8,999

E. Over ¥9,000

5. What is your age?

A. 17 or younger

B. 18–25 years old

C. 26–30 years old

D. 31–40 years old

E. 41–49 years old

F. 50 years or older

1. What is your highest level of education completed?

A. Primary school or below

B. High school or secondary specialized school

C. Bachelor's degree or associate degree

D. Postgraduate degree or higher

**II. Survey on the degree of property service enterprises’ demands for the smart community development**

The property service enterprises demand system for smart community development comprises three categories: community safety, livability services, and community governance. Based on your personal living experience, please indicate your need for the following services in your community. How would you rate your need for each service? (Please use the scale: 1 = strongly unnecessary, 2 = unnecessary, 3 = neutral, 4 = necessary, and 5 = strongly necessary)

**Table S2. Survey on the Degree of Property Service Enterprises’ Demands for Smart Community Development**

| Dimension | ID | Indicators | Score |
| --- | --- | --- | --- |
| Community Safety | Y1-1 | Digital community emergency preparedness plan |  |
|  | Y1-2 | Emergency plan implementing |  |
|  | Y1-3 | Propaganda and education of emergency safety |  |
|  | Y1-4 | Abnormal events recording |  |
|  | Y1-5 | Management and control of key parts |  |
|  | Y1-6 | Building monitoring |  |
|  | Y1-7 | Life channel facilities monitoring |  |
|  | Y1-8 | Floating population services |  |
|  | Y1-9 | Smart object monitoring facilities |  |
|  | Y1-10 | Public facilities monitoring |  |
|  | Y1-11 | Smart firefighting facilities |  |
|  | Y1-12 | Community safety inspection |  |
|  | Y1-13 | Emergency duty |  |
|  | Y1-14 | Intelligent emergency alert and forecasting |  |
|  | Y1-15 | Coordinated emergency response |  |
|  | Y1-16 | Emergency broadcast system |  |
|  | Y1-17 | Emergency rescue alarm |  |
|  | Y1-18 | Emergency supplies reserve |  |
|  | Y1-19 | Emergency command and dispatch |  |
|  | Y1-20 | Post-response community safety evaluation |  |
| Livability Services | Y2-1 | Community service center |  |
|  | Y2-2 | Community self-service terminals |  |
|  | Y2-3 | Community health services |  |
|  | Y2-4 | Community medical services |  |
|  | Y2-5 | Intelligent older adult care services |  |
|  | Y2-6 | Centralized reporting & maintenance system |  |
|  | Y2-7 | Recycling system for used things |  |
|  | Y2-8 | Smart childcare |  |
| Community Governance | Y3-1 | Community grid-based governance |  |
|  | Y3-2 | Collaborative community governance |  |
|  | Y3-3 | Smart environment monitoring |  |
|  | Y3-4 | Community population management |  |
|  | Y3-5 | Community vehicle management |  |
|  | Y3-6 | Community party affairs management |  |
|  | Y3-7 | Community volunteer management |  |
|  | Y3-8 | Housing management |  |
|  | Y3-9 | Integrated government service system |  |
|  | Y3-10 | Support services for vulnerable groups |  |
|  | Y3-11 | Dispute mediation and legal outreach(conflict regulation) |  |
|  | Y3-12 | Community cultural and recreational activities |  |
|  | Y3-13 | Centralized incident dispatch and monitoring |  |
|  | Y3-14 | Community alert broadcasting and statistics |  |
|  | Y3-15 | Multi-sectoral linkage |  |
|  | Y3-16 | Monitoring of special population groups |  |
|  | Y3-17 | Support for vulnerable groups |  |

Ⅲ. Your other suggestions for the smart community development:

**Supplementary File S4-3. Questionnaire on** **public administrators’ demands for smart community development**

Dear Sir/Madam:

Greetings! We warmly invite you to join our research initiative. Our team is currently conducting research on public administrators’ demands for the smart community development. To better understand your demands for smart community development, we would be most grateful for your valuable input. We hope you could take a few minutes from your busy schedule to complete this anonymous questionnaire. All responses will be kept confidential and used solely for statistical analysis. Your cooperation would be highly appreciated!

Institute of Engineering Management,

China University of Mining and Technology

**I. Basic Information**

1. What is your gender?

A. Male

B. Female

2. What is your age?

A. 22 or younger

B. 23-30 years old

C. 31-40 years old

D. 41-49 years old

E. 50 years or older

3. What is your highest education level?

A. Junior high school or below

B. High school or secondary specialized school

C. Bachelor's or associate degree

D. Postgraduate degree or higher

4. What is your political affiliation?

A. Member of the Communist Party of China or Probationary Member

B. Member of the Communist Youth League

C. Member of a democratic party

D. Non-affiliated individual

E. General public

5. How familiar are you with smart community services?

A. Very familiar

B. Familiar

C. Neutral

D. Unfamiliar

E. Very unfamiliar

6. What is your community management position?

A. Community Service Center Staff: Primarily responsible for daily operations including resident inquiries, cultural activities, and community events.

B. Community Grid Manager: Primarily responsible for grid-based management within the community, including patrols, service delivery, and administrative tasks.

C. Neighborhood Committee Member: Serves as a resident representative in community governance, responsible for voicing resident opinions, communication, and coordination.

D. Other

7. How many years of experience do you have in community management?

A. Less than 1 year

B. 1-3 years

C. 4-5 years

D. 5-10 years

E. More than 10 years

**II. Survey on the degree of public administrators’ demands for the smart community development**

The public administrators demand system for smart community development comprises three categories: community safety, livability services, and community governance. Based on your personal living experience, please indicate your need for the following services in your community. How would you rate your need for each service? (Please use the scale: 1 = strongly unnecessary, 2 = unnecessary, 3 = neutral, 4 = necessary, and 5 = strongly necessary)

**Table S3. Survey on the Degree of Public Administrators’ Demands for Smart Community Development**

| Dimension | ID | Indicators | Score |
| --- | --- | --- | --- |
| Community Safety | Y1-1 | Digital community emergency preparedness plan |  |
|  | Y1-2 | Emergency plan implementing |  |
|  | Y1-3 | Propaganda and education of emergency safety |  |
|  | Y1-4 | Abnormal events recording |  |
|  | Y1-5 | Management and control of key parts |  |
|  | Y1-6 | Building monitoring |  |
|  | Y1-7 | Life channel facilities monitoring |  |
|  | Y1-8 | Floating population services |  |
|  | Y1-9 | Smart object monitoring facilities |  |
|  | Y1-10 | Public facilities monitoring |  |
|  | Y1-11 | Smart firefighting facilities |  |
|  | Y1-12 | Community safety inspection |  |
|  | Y1-13 | Emergency duty |  |
|  | Y1-14 | Intelligent emergency alert and forecasting |  |
|  | Y1-15 | Coordinated emergency response |  |
|  | Y1-16 | Emergency broadcast system |  |
|  | Y1-17 | Emergency rescue alarm |  |
|  | Y1-18 | Emergency supplies reserve |  |
|  | Y1-19 | Emergency command and dispatch |  |
|  | Y1-20 | Post-response community safety evaluation |  |
| Livability Services | Y2-1 | Community service center |  |
|  | Y2-2 | Community self-service terminals |  |
|  | Y2-3 | Community health services |  |
|  | Y2-4 | Community medical services |  |
|  | Y2-5 | Intelligent older adult care services |  |
|  | Y2-6 | Centralized reporting & maintenance system |  |
|  | Y2-7 | Recycling system for used things |  |
|  | Y2-8 | Smart childcare |  |
| Community Governance | Y3-1 | Community grid-based governance |  |
|  | Y3-2 | Collaborative community governance |  |
|  | Y3-3 | Smart environment monitoring |  |
|  | Y3-4 | Community population management |  |
|  | Y3-5 | Community vehicle management |  |
|  | Y3-6 | Community party affairs management |  |
|  | Y3-7 | Community volunteer management |  |
|  | Y3-8 | Housing management |  |
|  | Y3-9 | Integrated government service system |  |
|  | Y3-10 | Support services for vulnerable groups |  |
|  | Y3-11 | Dispute mediation and legal outreach(conflict regulation) |  |
|  | Y3-12 | Community cultural and recreational activities |  |
|  | Y3-13 | Centralized incident dispatch and monitoring |  |
|  | Y3-14 | Community alert broadcasting and statistics |  |
|  | Y3-15 | Multi-sectoral linkage |  |
|  | Y3-16 | Monitoring of special population groups |  |
|  | Y3-17 | Support for vulnerable groups |  |

Ⅲ. Your other suggestions for the smart community development:

**Supplementary File S4-4. Questionnaire on** **social organizations’ demands for smart community development**

Dear Sir/Madam:

Greetings! We warmly invite you to join our research initiative. Our team is currently conducting research on social organizations’ demands for the smart community development. To better understand your demands for smart community development, we would be most grateful for your valuable input. We hope you could take a few minutes from your busy schedule to complete this anonymous questionnaire. All responses will be kept confidential and used solely for statistical analysis. Your cooperation would be highly appreciated!

Institute of Engineering Management,

China University of Mining and Technology

**I. Basic Information**

1. What is your gender?

A. Male

B. Female

2. What is your age?

A. 22 or younger

B. 23-30 years old

C. 31-40 years old

D. 41-49 years old

E. 50 or older

3. What is your position in the community social organization?

A. President/Chairperson/Vice-President/Vice-Chairperson

B. Secretary-General/Deputy Secretary-General

C. Department Head (e.g., Operations, Business, Finance, Human Resources)

D. Other

4. What type is your community social organization?

A. Volunteer Support Organization

B. Rights Protection & Services Organization

C. Daily Life Services Organization

D. Education & Training Organization

E. Charity & Public Welfare Organization

F. Cultural & Recreational Organization

G. Healthcare Organization

H. Other_______

5. How many years have you been working in community social organizations?

A. 1 year or less

B. 1-3 years

C. 4-5 years

D. More than 5 years

**II. Survey on the degree of social organizations’ demands for the smart community development**

The social organizations demand system for smart community development comprises three categories: community safety, livability services, and community governance. Based on your personal living experience, please indicate your need for the following services in your community. How would you rate your need for each service? (Please use the scale: 1 = strongly unnecessary, 2 = unnecessary, 3 = neutral, 4 = necessary, and 5 = strongly necessary)

**Table S4. Survey on the Degree of Social Organizations’ Demands for Smart Community Development**

| Dimension | ID | Indicators | Score |
| --- | --- | --- | --- |
| Community Safety | Y1-1 | Digital community emergency preparedness plan |  |
|  | Y1-2 | Emergency plan implementing |  |
|  | Y1-3 | Propaganda and education of emergency safety |  |
|  | Y1-4 | Abnormal events recording |  |
|  | Y1-5 | Management and control of key parts |  |
|  | Y1-6 | Building monitoring |  |
|  | Y1-7 | Life channel facilities monitoring |  |
|  | Y1-8 | Floating population services |  |
|  | Y1-9 | Smart object monitoring facilities |  |
|  | Y1-10 | Public facilities monitoring |  |
|  | Y1-11 | Smart firefighting facilities |  |
|  | Y1-12 | Community safety inspection |  |
|  | Y1-13 | Emergency duty |  |
|  | Y1-14 | Intelligent emergency alert and forecasting |  |
|  | Y1-15 | Coordinated emergency response |  |
|  | Y1-16 | Emergency broadcast system |  |
|  | Y1-17 | Emergency rescue alarm |  |
|  | Y1-18 | Emergency supplies reserve |  |
|  | Y1-19 | Emergency command and dispatch |  |
|  | Y1-20 | Post-response community safety evaluation |  |
| Livability Services | Y2-1 | Community service center |  |
|  | Y2-2 | Community self-service terminals |  |
|  | Y2-3 | Community health services |  |
|  | Y2-4 | Community medical services |  |
|  | Y2-5 | Intelligent older adult care services |  |
|  | Y2-6 | Centralized reporting & maintenance system |  |
|  | Y2-7 | Recycling system for used things |  |
|  | Y2-8 | Smart childcare |  |
| Community Governance | Y3-1 | Community grid-based governance |  |
|  | Y3-2 | Collaborative community governance |  |
|  | Y3-3 | Smart environment monitoring |  |
|  | Y3-4 | Community population management |  |
|  | Y3-5 | Community vehicle management |  |
|  | Y3-6 | Community party affairs management |  |
|  | Y3-7 | Community volunteer management |  |
|  | Y3-8 | Housing management |  |
|  | Y3-9 | Integrated government service system |  |
|  | Y3-10 | Support services for vulnerable groups |  |
|  | Y3-11 | Dispute mediation and legal outreach(conflict regulation) |  |
|  | Y3-12 | Community cultural and recreational activities |  |
|  | Y3-13 | Centralized incident dispatch and monitoring |  |
|  | Y3-14 | Community alert broadcasting and statistics |  |
|  | Y3-15 | Multi-sectoral linkage |  |
|  | Y3-16 | Monitoring of special population groups |  |
|  | Y3-17 | Support for vulnerable groups |  |

Ⅲ. Your other suggestions for the smart community development:
