# Supplementary material for: Decoding stakeholders' demand to map the future of smart communities: evidence from China
Source: Front Public Health. 2026 Mar 13;14:1751235. doi: 10.3389/fpubh.2026.1751235 (PMC13021643; doi:10.3389/fpubh.2026.1751235)
Supplement: Supplementary file 5 [file Table_5.docx]

Supplementary File S5

**Table S6. The full names and coding details of surveyed smart communities**

| **City** | **ID** | **The full name of surveyed smart community** | **Corresponding code** |
| --- | --- | --- | --- |
| Dongguan | 1 | Batou community | C1 |
|  | 2 | Hongtu community | C2 |
|  | 3 | Baoshi community | C3 |
|  | 4 | Shimei community | C4 |
|  | 5 | Wanjiang community | C5 |
|  | 6 | Fuhua community | C6 |
| Huizhou | 7 | Banzhangling community | C7 |
|  | 8 | Qingxi community | C8 |
|  | 9 | Siqian community | C9 |
|  | 10 | Tangjing community | C10 |
|  | 11 | Tongxin community | C11 |
| Luoyang | 12 | Chaoyang community | C12 |
|  | 13 | Letian community | C13 |
|  | 14 | Xiguan community | C14 |
|  | 15 | Xiyaun community | C15 |
|  | 16 | Xinglong community | C16 |
| Putian | 17 | Beimo community | C17 |
|  | 18 | Fengshan community | C18 |
|  | 19 | Longqiao community | C19 |
|  | 20 | Yinglong community | C20 |
|  | 21 | Yunei community | C21 |
| Shenzhen | 22 | Baolong community | C22 |
|  | 23 | Fuguang community | C23 |
|  | 24 | Huilongpu community | C24 |
|  | 25 | Nanyuan community | C25 |
|  | 26 | Yucun community | C26 |
| Zhengzhou | 27 | Dongfenglu community | C27 |
|  | 28 | Haichuan community | C28 |
|  | 29 | Jinghu community | C29 |
|  | 30 | Kaiyuan community | C30 |
|  | 31 | Longjiangbeili community | C31 |
|  | 32 | Yingbinlu community | C32 |
