# Supplementary material for: Decoding stakeholders' demand to map the future of smart communities: evidence from China
Source: Front Public Health. 2026 Mar 13;14:1751235. doi: 10.3389/fpubh.2026.1751235 (PMC13021643; doi:10.3389/fpubh.2026.1751235)
Supplement: Supplementary file 6 [file Table_6.docx]

Supplementary File S6

The complete statistics of respondents

**Table S7. Descriptive statistics of residents’ characteristics**

| **Respondent characteristics** | **Node** | **Items** | | **Frequency** | **Percentage（N=1132）** |
| --- | --- | --- | --- | --- | --- |
| X1：  Demographic characteristics | x11 | Gender | Male | 560 | 49.47% |
|  |  |  | Female | 572 | 50.53% |
|  | x12 | Age | 17 or younger | 14 | 1.24% |
|  |  |  | 18-25 years old | 406 | 35.87% |
|  |  |  | 26-30 years old | 256 | 22.61% |
|  |  |  | 31-40 years old | 373 | 32.95% |
|  |  |  | 41-49 years old | 67 | 5.92% |
|  |  |  | 50 or older | 16 | 1.41% |
|  | x13 | Highest level of education | Primary school or below | 4 | 0.35% |
|  |  |  | Junior high school | 30 | 2.65% |
|  |  |  | High school or secondary specialized school | 102 | 9.01% |
|  |  |  | Associate degree | 194 | 17.14% |
|  |  |  | Bachelor’s degree | 754 | 66.61% |
|  |  |  | Postgraduate degree or higher | 48 | 4.24% |
| X2：  Residential characteristics | x21 | Length of residence | Less than 1 year | 145 | 12.81% |
|  |  |  | 1 to 3 years | 270 | 23.85% |
|  |  |  | More than 3 years | 717 | 63.34% |
| X3：  Economic characteristics | x31 | Average monthly disposable income | Under ¥3,000 | 250 | 22.08% |
|  |  |  | ¥3,000 - ¥4,999 | 249 | 22% |
|  |  |  | ¥5,000 - ¥6,999 | 219 | 19.35% |
|  |  |  | ¥7,000 - ¥8,999 | 180 | 15.9% |
|  |  |  | Over ¥9,000 | 234 | 20.67% |
| X4：  Cognitive characteristics | x41 | Familiarity with smart community services | Very familiar | 149 | 13.16% |
|  |  |  | Familiar | 422 | 37.28% |
|  |  |  | Neutral / Somewhat familiar | 420 | 37.1% |
|  |  |  | Unfamiliar | 111 | 9.81% |
|  |  |  | Very unfamiliar | 30 | 2.65% |

**Table S8. Descriptive statistics of property service enterprises’ characteristics**

| **Respondent characteristics** | **Node** | **Items** | | **Frequency** | **Percentage（N=262）** |
| --- | --- | --- | --- | --- | --- |
| X1：  Characteristics of the property service enterprises | x11 | Number of households under management | 1,000 households or fewer | 36 | 13.74% |
|  |  |  | 1,000–1,999 households | 107 | 40.84% |
|  |  |  | 2,000–2,999 households | 70 | 26.72% |
|  |  |  | 3,000–3,999 households | 37 | 14.12% |
|  |  |  | 4,000 households or more | 12 | 4.58% |
|  | x12 | Number of employees stationed in the community | 20 or fewer | 19 | 7.25% |
|  |  |  | 21–40 employees | 90 | 34.35% |
|  |  |  | 41–60 employees | 62 | 23.66% |
|  |  |  | 61–80 employees | 40 | 15.27% |
|  |  |  | 81–100 employees | 24 | 9.16% |
|  |  |  | More than 100 employees | 27 | 10.31% |
| X2：  Characteristics of property service personnel | x21 | Years of work experience in property service enterprises | 1 year or less | 12 | 4.58% |
|  |  |  | 1–3 years | 37 | 14.12% |
|  |  |  | 4–5 years | 75 | 28.63% |
|  |  |  | More than 5 years | 138 | 52.67% |
|  | x22 | Average monthly income | Under ¥3,000 | 5 | 1.91% |
|  |  |  | ¥3,000 - ¥4,999 | 33 | 12.60% |
|  |  |  | ¥5,000 - ¥6,999 | 85 | 32.44% |
|  |  |  | ¥7,000 - ¥8,999 | 88 | 33.59% |
|  |  |  | Over ¥9,000 | 51 | 19.47% |
|  | x23 | Age | 17 or younger | 0 | 0% |
|  |  |  | 18–25 years old | 17 | 6.49% |
|  |  |  | 26–30 years old | 59 | 22.52% |
|  |  |  | 31–40 years old | 150 | 57.25% |
|  |  |  | 41–49 years old | 30 | 11.45% |
|  |  |  | 50 years or older | 6 | 2.29% |
|  | x24 | Highest level of education | Primary school or below | 7 | 2.67% |
|  |  |  | High school or secondary specialized school | 43 | 16.41% |
|  |  |  | Bachelor’s degree or associate degree | 206 | 78.63% |
|  |  |  | Postgraduate degree or higher | 6 | 2.29% |

**Table S9. Descriptive statistics of public administrators’ characteristics**

| **Respondent characteristics** | **Node** | **Items** | | **Frequency** | **Percentage（N=103）** |
| --- | --- | --- | --- | --- | --- |
| X1：  Demographic characteristics | x11 | Gender | Male | 52 | 50.49% |
|  |  |  | Female | 51 | 49.51% |
|  | x12 | Age | 22 or younger | 0 | 0.00% |
|  |  |  | 23-30 years old | 18 | 17.48% |
|  |  |  | 31-40 years old | 79 | 76.70% |
|  |  |  | 41-49 years old | 6 | 5.83% |
|  |  |  | 50 years or older | 0 | 0.00% |
|  | x13 | Highest level of education | Primary school or below | 0 | 0.00% |
|  |  |  | High school or secondary specialized school | 4 | 3.88% |
|  |  |  | Bachelor's degree or associate degree | 95 | 92.23% |
|  |  |  | Postgraduate degree or higher | 4 | 3.88% |
|  | x14 | Political affiliation | Member of the Communist Party of China or Probationary Member | 74 | 71.84% |
|  |  |  | Member of the Communist Youth League | 14 | 28.96% |
|  |  |  | Member of a democratic party | 3 | 2.91% |
|  |  |  | Non-affiliated individual | 0 | 0.00% |
|  |  |  | General public | 12 | 11.65% |
| X2：  Cognitive characteristics | x21 | Familiarity with smart community services | Very familiar | 24 | 23.30% |
|  |  |  | Familiar | 71 | 68.93% |
|  |  |  | Neutral / Somewhat familiar | 8 | 7.77% |
|  |  |  | Unfamiliar | 0 | 0.00% |
|  |  |  | Very unfamiliar | 0 | 0.00% |
| X3：  Job characteristics | x31 | Community management position | Community Service Center Staff: Primarily responsible for daily operations including resident inquiries, cultural activities, and community events. | 55 | 53.40% |
|  |  |  | Community Grid Manager: Primarily responsible for grid-based management within the community, including patrols, service delivery, and administrative tasks. | 36 | 34.95% |
|  |  |  | Neighborhood Committee Member: Serves as a resident representative in community governance, responsible for voicing resident opinions, communication, and coordination. | 12 | 11.65% |
|  |  |  | Other | 0 | 0.00% |
|  | x32 | years of experience in community management | Less than 1 year | 2 | 1.94% |
|  |  |  | 1-3 years | 14 | 13.59% |
|  |  |  | 4-5 years | 36 | 34.95% |
|  |  |  | 5-10 years | 43 | 41.75% |
|  |  |  | More than 10 years | 8 | 7.77% |

**Table S10. Descriptive statistics of social organizations’ characteristics**

| **Respondent characteristics** | **Node** | **Items** | | **Frequency** | **Percentage（N=109）** |
| --- | --- | --- | --- | --- | --- |
| X1：  Demographic characteristics | x11 | Gender | Male | 43 | 39.45% |
|  |  |  | Female | 66 | 60.55% |
|  | x12 | Age | 22 or younger | 0 | 0% |
|  |  |  | 23-30 years old | 27 | 24.77% |
|  |  |  | 31-40 years old | 80 | 73.39% |
|  |  |  | 41-49 years old | 2 | 1.83% |
|  |  |  | 50 years or older | 0 | 0% |
| X2：  Job characteristics | x21 | Position in the community social organization | President/Chairperson/Vice-President/Vice-Chairperson | 1 | 0.92% |
|  |  |  | Secretary-General/Deputy Secretary-General | 9 | 8.26% |
|  |  |  | Department Head (e.g., Operations, Business, Finance, Human Resources) | 97 | 88.99% |
|  |  |  | Other | 2 | 1.83% |
|  | x22 | Type of community social organization | Volunteer Support Organization | 22 | 20.18% |
|  |  |  | Rights Protection & Services Organization | 6 | 5.50% |
|  |  |  | Daily Life Services Organization | 37 | 33.94% |
|  |  |  | Education & Training Organization | 17 | 15.60% |
|  |  |  | Charity & Public Welfare Organization | 11 | 10.09% |
|  |  |  | Cultural & Recreational Organization | 9 | 8.26% |
|  |  |  | Healthcare Organization | 7 | 6.42% |
|  |  |  | Other | 0 | 0% |
|  | x23 | Years of experience in community social organizations | 1 year or less | 0 | 0% |
|  |  |  | 1-3 years | 10 | 9.17% |
|  |  |  | 4-5 years | 26 | 23.85% |
|  |  |  | More than 5 years | 73 | 66.97% |
